# Supplementary material for: Innovative strategies and implementation science approaches for health delivery among migrants in humanitarian settings: A scoping review
Source: PLOS Glob Public Health. 2024 Dec 2;4(12):e0003514. doi: 10.1371/journal.pgph.0003514 (PMC11611092; doi:10.1371/journal.pgph.0003514)
Supplement: S1 Appendix — This table presents all the extracted data from studies included in the review including study rigor, descriptors, determinants, strategies, location, and use of implementation science theories, models, or frameworks for each study. (DOCX) [file pgph.0003514.s001.docx]

S1 Appendix: Comprehensive data extraction from scoping review studies for displaced migrants in humanitarian settings

| Authors | Study title | Year | Location (country) | Methods | Topic area | Key findings | Rigor | Identification of determinants | Implementation strategies | IS frameworks | Quantitative scope | Health outcomes |
| --- | --- | --- | --- | --- | --- | --- | --- | --- | --- | --- | --- | --- |
| Abdullahi et al. | Providing TB and HIV outreach services to internally displaced populations in Northeast Nigeria: Results of a controlled intervention study | 2020 | Nigeria | Pre/post intervention study with interrupted time series analysis | Infectious disease | Implemented TB case finding intervention in 26 IDP camps leading to 283,556 screenings | 3 | Facilitators: local partnership, political priorities  Barriers: health worker shortage, low resources, security risks | Needs assessment, stakeholder engagement, capacity building, established referral pathways | No | Yes | Yes |
| Ahmed et al. | Burden of eye disease and demand for care in the Bangladesh Rohingya displaced population and host community: A cohort study | 2020 | Bangladesh | Cross-sectional cohort study | Ophthalmology | Blindness and prevalence requiring cataract surgery among Rohingya refugees is greater than host population | 6 | Facilitators: Political support, health worker availability, local partners  Barriers: none | Targeted outreach, inclusion of host communities, mobile clinics, information dissemination | No | Yes | No |
| Akhtar et al. | Feasibility trial of a scalable transdiagnostic group psychological intervention for Syrians residing in a refugee camp | 2021 | Jordan | Feasibility randomized control trial | Mental health | Among 64 participants, the task-shifting psychosocial program GroupPM+ was shown to be feasible and culturally acceptable in the camp setting for Syrian refugees and showed reductions in psychological distress symptoms of participants’ children. | 4 | Facilitators: Positive perceptions towards the intervention  Barriers: Decreased engagement from outset, recruitment script issues, participants returning to Syria | Feedback mechanisms, local collaboration, political priorities, task shifting | No | Yes | Yes |
| Alemu et al. | Health and nutrition emergency response among internally displaced persons at Ranch collective site, Chagni, Ethiopia: The role of emergency operation center, lessons from the field, and way forwards | 2022 | Ethiopia | Case study | Primary care, mental health | The Public Health Emergency Operation Center provided free essential health services to 33,410 IDPs including mental health, trachomatous trichiasis, cataract surgery, antenatal care, birth attendance, COVID-19 vaccination, and malnutrition services | 6 | Facilitators: Clear and standard procedures, High-income institution or other international partnership  Barriers: Health worker shortage, funding restrictions, resource limitations, environmental conditions, lack of expertise | Political priorities, centralized coordination, needs assessment, collaborating with local partners, feedback mechanisms, stakeholder engagement, accurate data monitoring, supervision, information dissemination and community education | No | Yes | No |
| Ali et al. | The role of integrated programs in the prevention of COVID-19 in a humanitarian setting | 2022 | Sudan | Case study | Infectious Disease | The interventional program aimed at early preparedness, including proper training, coordination and follow-up, has significantly contributed to limiting the spread of COVID-19 in South Korfodon | 6 | Facilitators: availability of health workers, monitoring systems  Barriers: No | Capacity building, stakeholder analysis, feedback, iterative support | No | Yes | No |
| Amani et al. | Preventive mass vaccination campaign against meningococcal meningitis in refugee camps of Cameroon during the COVID-19 pandemic: vaccination coverage, challenges, best practices, and lessons learned | 2021 | Cameroon | Cross-sectional | Infectious disease | Preventive mass vaccination against meningococcal meningitis was successful. | 6 | Facilitators: Communication, involvement of local leaders  Barriers: misconceptions that this was a COVID-19 vaccine | Capacity building, involving community leaders, feedback mechanisms, vaccine checklist cards | No | Yes | No |
| Amsalu et al. | Lessons learned from helping babies survive in humanitarian settings | 2020 | Somalia, Niger, Chad, Cameroon | Pre/post descriptional study | Child health | Implementation of helping babies survive at 3 refugee settings | 4 | Facilitators: political priorities, program adaptability  Barriers: shortage of health workers, low knowledge/literacy, security risks, resource limitations | Train-the-trainer, capacity building, needs assessment, ongoing support, feedback mechanisms | No | Yes | No |
| Ansbro et al. | Fixed dose combination drugs for  cardiovascular disease in a prolonged  humanitarian crisis in Lebanon: an  implementation study | 2023 | Lebanon | Prospective pe-post implementation study | Non-communicable disease | 418 Syrian refugee participants switched to fixed dose combination therapy medications for atherosclerotic cardiovascular disease secondary prevention | 4 | Facilitators: Clear and standard procedures, established resource supply chains, intervention effectiveness, low-cost, alignment with political priorities  Barriers: physical space closure | Capacity building, centralized coordination, integration into existing health systems | No | Yes | Yes |
| Ansbro et al. | MSF experiences of providing multidisciplinary primary level NCD care for Syrian refugees and the host population in Jordan: an implementation study guided by the RE-AIM framework | 2021 | Jordan | Cohort implementation study | Noncommunicable disease | A multi-disciplinary, primary-level model of NCD care for Syrian refugees in Jordan, when evaluated with the RE-AIM framework, demonstrated good clinical outcomes for limited patients and at high cost. | 4 | Facilitators: trusted, good quality care in a caring environment, solution-focused approaches and motivational interviewing techniques  Barriers: indirect costs of clinical attendance (transport, lost work time), trauma from conflict limiting engagement, inadequate low-cost referral options, challenges for MSF to rapidly adapt to a highly complex health system, use of combative health education delivery | Appointment-based consultations supported by a local management team and coordination team in Amman | RE-AIM | Yes | Yes |
| Ayoya et al. | Protecting and improving breastfeeding practices during a major emergency: lessons learnt from the baby tents in Haiti | 2013 | Haiti | Case study | Child health | 193 baby tents cared for 180,499 mother-infant pairs to limit breastfeeding interruptions from the 2010 earthquake | 6 | Facilitators: available health workers  Barriers: displacement of people post-earthquake, determining how many infants needed which formula, sustainability of the program, pre-existing community beliefs on breastfeeding, weakened centralized health ministry, resource constraints | Capacity building, stakeholder engagement, educational materials made in local language, multidisciplinary teams, information dissemination | No | Yes | No |
| Azad et al. | Breastfeeding support through wet nursing during nutritional emergency: A cross sectional study from Rohingya refugee camps in Bangladesh | 2019 | Bangladesh | Cross-sectional | Child health | Wet nurses can be a valuable addition to an IYCF-E protocol. | 6 | Facilitators: Wet nurses’ willingness to volunteer, community counseling, political support, religious inspiration  Barriers: health worker shortages, lack of knowledge of wet nursing by refugees, time limitations, travel distance, poor family compliance | No | No | No | No |
| Barua et al. | Implementation of a community-based referral project to improve access to emergency obstetric and newborn care in Rohingya population during COVID-19 pandemic in Bangladesh | 2022 | Bangladesh | Case study | Maternal health | 3,330 referrals were made through a community-based transport intervention to address second delay among emergency obstetric patients | 6 | Facilitators: health worker availability, intervention free to population, trust in health workers, 24/7 service availability  Barriers: resource limitations, geographic barriers, COVID-19 pandemic, poor network connectivity | Establishing infrastructure or designating specialized physical space, needs assessment, collaborating with local partners, supervision, information dissemination and community education, capacity building, local champions | No | Yes | No |
| Bastin et al. | Description and predictive factors of individual outcomes in a refugee camp based mental health intervention (Beirut, Lebanon) | 2013 | Lebanon | Retrospective cohort | Mental health | Mental health program among 1144 patients showed improved functionality and need for adaptation to more severe and less educated patients | 6 | Facilitators: Positive perceptions of intervention, pre-existing partnership with religious authorities  Barriers: Not optimized for severe mental illness, lack of follow-up from migration, low data quality quality (too much information), low health literacy | Stakeholder engagement, information dissemination | No | Yes | Yes |
| Beeman et al. | Using human-centered design to codesign dedicated menstrual health spaces  with people who menstruate in Bidi Bidi  refugee settlement, Uganda: Learnings  for further adaptation and scale  in humanitarian settings | 2023 | Uganda | Case study | Sexual and reproductive health | The Cocoon Mini, an intervention to facilitate menstrual health, showed high desirability and acceptability among 175 people who menstruate and community members. | 6 | Facilitators: adaptability of intervention, high-income institution partnership, positive perceptions towards intervention  Barriers: lack of evidence base, resource limitations, environmental conditions, security risks, resource limitations, geographic barriers, financial imitations, sex disparities | Piloting, needs assessment, collaborating with local partners, intervention adaptation, information dissemination, local champions, establishing infrastructure, group activities, feedback mechanisms | No | Yes | No |
| Beshr et al. | Polio outbreak response, Yemen | 2023 | Yemen | Case study | Infectious disease | 7.2 million children vaccinated for polio in response to outbreaks in conflict-affected areas and multiple sanitary measures implemented in IDP camps | 4 | Facilitators: pre-existing partnerships, high-income country partnership, funding availability, alignment with political priorities  Barriers: security risks, population movement, COVID-19 lockdown, negative perceptions towards intervention, lack of government presence, environmental conditions | Collaboration with international partners, information dissemination, advocacy, social mobilization, local champions, local partnerships, technology integration | No | Yes | Yes |
| Bile et al. | Protecting the right to health of internally displaced mothers and children: the imperative of inter-cluster coordination for translating best practices into effective participatory action | 2011 | Pakistan | Case study | Primary care | Pakistani government and WHO collaborated to organize a health cluster of 46 humanitarian partners to reinforce health system following natural disaster | 6 | Facilitators: Mobilization of female physicians and those who spoke local language, incentive-based approach for health education  Barriers: Funds | Involvement of community members, capacity building, piloting programs before scaling, interdisciplinary approaches | No | No | No |
| Bile, et al. | Learning through crisis: development and implementation of a health cluster strategy for internally displaced persons | 2010 | Pakistan | Case study | Primary care | Implementation of the health cluster disease warning system was key in controlling disease outbreaks. The essential health service package ensured delivery of primary health care services and emergency interventions. | 6 | Facilitators: implementation of disease early warning system, participation strategy formation, established national center for health emergency preparedness  Barriers: large number of IDPs with dispersed presence, communicable disease risk, health personnel shortage | Needs assessment, technology integration, EBI bundling, feedback mechanisms, supervision | Humanitarian health response coordination framework | Yes | No |
| Blackwell et al. | Sustaining psychotherapist effectiveness and independence: An exploratory study with displaced persons in Kurdistan, Iraq | 2022 | Iraq | Cohort implementation study | Mental health | Local psychotherapists were shown to decrease PTSD and depression scores among 28 displaced persons | 4 | Facilitators: acceptance of mental health services and receiving psychotherapy  Barriers: non-randomized, time and resource scarcity, low data quality | Capacity building by training health worker, involving community workers | No | Yes | Yes |
| Bolton, et al | Interventions for depression symptoms among adolescent survivors of war and displacement in Northern Uganda: A randomized controlled trial | 2007 | Uganda | Randomized controlled trial | Mental health | 314 adolescent girls who received group interpersonal psychotherapy showed significant improvement in depression symptoms compared to controls whereas improvement in boys was not significant . Creative play had no effect on depression severity | 2 | Facilitators: previous experience, close NGO partnership, local population embracing methodology, local culture and language implementation  Barriers: difficulty with cultural competency, literacy rates, cultural expectations | Stakeholder engagement,, capacity building, feedback mechanism, needs assessment adapting program to local context | No | Yes | Yes |
| Borja et al.. | Child-centered, cross-sectoral mental health and psychosocial support interventions in the Rohingya response: A field report by Save the Children | 2019 | Bangladesh | Case study | Mental health | Mental health and psychosocial services should be cross-sectoral and consistent, and must be prioritized by local agents. | 6 | Facilitators: Positive perceptions of interventions, technical capacity of personnel  Barriers: Change in staff and shortage of health workers, evolution of the crisis, physical barriers to access | No | Intervention mapping | No | No |
| Bosqui et al. | What drives change in children receiving telephone-delivered Common Elements Treatment Approach (t-CETA)? A multiple n = 1 study with Syrian refugee children and adolescents in Lebanon | 2023 | Lebanon | Multiple n=1 implementation design | Mental health | Among 9 Syrian refugee children, those with war-related trauma were more likely to show improvement across symptom depression symptom clusters | 3 | Facilitators: evidence base, language and cultural adaptation, social interconnectedness, familial support  Barriers: none | Local champions, supervision, piloting, audits | No | Yes | Yes |
| Bryant et al. | Effectiveness of a brief group behavioral intervention for common mental disorders in Syrian refugees in Jordan: A randomized controlled trial | 2022 | Jordan | Randomized control trial | Mental health | Among 410 refugees, lay-provider group-based psychological intervention yields positive results for depressive symptoms, self-awareness, and parenting approach | 2 | Facilitators: Political situation leading to low attrition, facilitator availability, intervention scalability  Barriers: Limited applicability to PTSD, disability, or children’s psych issues. Trial “fatigue” at the 3 mo mark in experimental arm | Local champions | No | Yes | Yes |
| Bryant et al. | Effectiveness of a brief group behavioural intervention on psychological distress in young adolescent Syrian refugees: A randomised controlled trial | 2022 | Jordan | Randomized control trial | Mental health | Syrian refugees aged 10-14 showed reduced internalization of problems following the EASE intervention. Caregivers had reduced distress and more consistent disciplinary styles. | 2 | Facilitators: Minimal training for intervention, political environment facilitating implementation  Barriers: COVID-19 lockdowns increased participant distress, reliance on metrics that are unvalidated among Arabic adolescents, sex disparities | Local champions, capacity building | No | Yes | Yes |
| Bryant et al. | Twelve-month follow-up of a randomised clinical trial of a brief group psychological intervention for common mental disorders in Syrian refugees in Jordan | 2022 | Jordan | Randomized control trial | Mental health | gPM+ was implemented among 410 Syrian refugees and demonstrated greater reductions in depression at 3 months but 1 year. | 2 | Facilitators: none  Barriers: limited long-term intervention evidence, security risks, poverty | Task shifting, local collaboration, capacity building, supervision, remuneration | No | Yes | Yes |
| Carrara et al | Longer exposure to new refugee food ration is associated with reduced prevalence of small for gestational age: results from 2 cross-sectional surveys on the Thailand-Myanmar border | 2017 | Thailand | Quasi-experimental cohort implementation study | Maternal and child health | Among 987 newborns, introducing micronutrient fortified flour (MFF) in the diet of pregnant refugee women improved outcomes for newborns | 3 | Facilitators: routine antenatal care, high service uptake, education and demonstrations  Barriers: low data quality, lack of reliability for intervention use | Needs assessment, collaboration with local stakeholders, information sessions, capacity building, routine monitoring, feedback mechanism | No | Yes | Yes |
| Centers for Disease Control | Emergency measles control activities--Darfur, Sudan, 2004 | 2004 | Sudan | Case study | Infectious disease | Mass measles vaccination campaigns in IDP camps in Darfur proved largely successful leading to significant reduction in measles cases | 6 | Facilitators: Negotiations with rebel forces, population open to vaccination  Barriers: security risks, inaccessible regions (West Darfur) due to exodus, rainy season, supply chain difficulties | Local champions capacity building, local and international collaboration, interdisciplinary approach | No | Yes | Yes |
| Chowdhury et al. | A formative research to explore the  programmatic approach of vaccinating the  Rohingya refugees and host communities  against COVID-19 infection in Bangladesh | 2023 | Bangladesh | Case study | Infectious disease | Implemented COVID-19 vaccination campaign among Rohingya refugees and examining vaccine hesitancy and determinants to project success. | 6 | Facilitators: high-income country partnership, communication systems  Barriers: negative perceptions of the intervention, funding restrictions, staff motivation, resource insufficiencies, low health literacy, cost, social prejudice, lack of cultural appropriateness | Establishing infrastructure and resource supply chains, stakeholder engagement, feedback mechanisms, information dissemination, integrating technology | No | No | No |
| Cohen et al. | Task-shifting for refugee mental health and psychosocial support: A scoping review of services in humanitarian settings through the lens of RE-AIM | 2021 | LMICs | Scoping review | Mental health | Among 11 studies, RE-AIM was used to assess adaptation strategies, feasibility, and fidelity, but there is a dearth of evidence on capacity building of lay providers | 5 | Facilitators: buy-in from community members  Barriers: high staff turnover, rapid migration of population, lack of sustainable funding | Health worker training, supervision, needs assessment, train-the-trainer, local champions, community autonomy, stakeholder involvement, routine monitoring, checklists | RE-AIM | Yes | No |
| Coldiron et al | Intermittent preventive treatment for malaria among children in a refugee camp in Northern Uganda: lessons learned | 2017 | Uganda | Quasi-experimental cohort study | Infectious diseases | Intermittent preventive treatment for malaria among 40,611 participants resulted in decreased incidence of malaria, high coverage in program implementation, strong safety profile, while parasite burden was not alleviated significantly among adults. | 3 | Facilitators: use of existing systems, community-based focus  Barriers: logistical difficulty, inefficiency of height-based dosing, non-participation, parasite reservoir in adults, intervention timing | Stakeholder engagement, surveillance, health record usage, health care worker training, voluntary participation, systematic sampling | No | Yes | Yes |
| Corna et al. | Supporting maternal mental health of Rohingya refugee women during the perinatal period to promote child health and wellbeing: a field study in Cox's Bazar | 2019 | Bangladesh | Case study | Maternal health | Among 260 pregnant women, psychosocial intervention (PSI) was found to be effective despite the chronic stressful conditions of the refugees in the camps | 6 | Facilitators: social workers have good psychosocial background, practical competencies and regular clinical supervision, expert clinical psychologists  Barriers: field workers research competencies, and time and budget restrictions | Community feedback mechanisms, PSI implemented at home visits and women support groups, local champions, monthly check-ins | No | Yes | Yes |
| Crombach et al | Impact and cultural acceptance of the narrative exposure therapy in the aftermath of a natural disaster in Burundi | 2018 | Burundi | Cohort impelentation | Mental health | Among 51 individuals, NET therapy improved PTSD and depressed symptoms | 3 | Facilitators: availability of local volunteers and counselors  Barriers: stigma, mistrust towards psychologists, misunderstandings about ideas including witchcraft, confusion about psychological interventions, loss of follow up | Collaborate with local stakeholders, use of local counselors, training local counselors, psychoeducation, feedback, needs assessment, capacity building | No | Yes | Yes |
| Curry et al. | Delivering high-quality family planning services in crisis-affected settings I: program implementation | 2015 | Chad, DRC, Djibouti, Mali, Pakistan | Case study | Sexual and reproductive health | SAFPAC successfully reached 52,616 new users of modern contraceptive methods | 6 | Facilitators: Pre-established health system, stock threshold-based resupply methods, support from local leaders  Barriers: travel constraints due to security concerns, limited trained health workers, resource limitations | Supervision, monthly checklists, feedback mechanisms, improved supply chain delivery, stakeholder engagement, capacity building, information dissemination, local champions | No | Yes | No |
| Curry et al. | Delivering high-quality family planning services in crisis-affected settings II: results | 2015 | Chad, DRC, Djibouti, Mali, Pakistan | Case study | Sexual and reproductive health | Same as above | 6 | Facilitators: Health worker availability  Barriers: Active conflict, natural disasters | Same as above | No | Yes | No |
| del Cacho et al. | Pharmaceutical humanitarian assistance in the establishment of a drug compounding laboratory in a refugee camp | 2021 | Algeria | Case study | Pharmacy | Successful establishment of a pharmacy distribution and training program | 6 | Facilitators: high-income institution partnership, specialized health worker availability  Barriers: extreme weather, limited resources | No | No | Yes | No |
| Donkeng-Donfack et al. | A cost-benefit algorithm for rapid diagnosis of tuberculosis and rifampicin resistance detection during mass screening campaigns | 2022 | Cameroon | Cost-benefit analysis | Infectious disease | The use of TB-LAMP as an initial diagnostic tool followed by Xpert MTB/RIF for diagnosing TB during mass campaigns has a cost benefit after 1 year of use. | 6 | Facilitators: Decreased turnaround time for testing  Barriers: Resource limitations | No | No | Yes | No |
| Dozio et al. | Baby friendly spaces: an intervention for pregnant and lactating women and their infants in Cameroon | 2020 | Cameroon | Cohort implementation study | Maternal / child health | Baby-friendly spaces support and improve the overall well-being of pregnant and lactating women in refugee camps by increasing likelihood of lactation and decreasing maternal stress | 3 | Facilitators: none  Barriers: Cultural trepidations in speaking about pregnancy | Trained professionals, utilizing existing referral systems, physical space establishment, intervention adaptation, interdisciplinary approach | No | Yes | Yes |
| Dyer et al. | Psychological and psychiatric care for Rohingya refugees in Bangladesh | 2019 | Bangladesh | Case study | Mental health | There is a need for a dedicated service to treat people with moderate to severe mental health disorders in a humanitarian context | 6 | Facilitators: pre-existing partnerships  Barriers: teams adapted to existing structures, health worker availability cultural factors around mental health, privacy and confidentiality | Needs assessment, capacity building, stakeholder engagement, task shifting | No | Yes | No |
| Ehiri et al. | Training and deployment of lay refugee/internally displaced persons to provide basic health services in camps: a systematic review | 2014 | Guinea, Tanzania, Belize, and Myanmar | Systematic review | Primary care | Training lay refugees as health workers had a positive impact on population health including increased service coverage, knowledge about disease symptoms and prevention, adoption of improved treatment seeking and protective behaviors, uptake of services, and access to reproductive health information | 5 | Facilitators: local cultural knowledge, community empowerment especially among women and children  Barriers: difficult to commit limited resources to planning and implementing rigorous evaluation | Capacity building, task-shifting, local autonomy | No | Yes | No |
| Eisenberg et al. | Diphtheria Antitoxin Administration, Outcomes, and Safety: Response to a Diphtheria Outbreak in Cox's Bazar, Bangladesh | 2021 | Bangladesh | Retrospective cohort | Infectious Disease | Among 709 patients, DAT is safe/feasible in low resource settings as long as several safety standards of monitoring/staff training/safety awareness expectations are met. | 6 | Facilitators: health worker availability, pre-established health system  Barriers: No standardized worldwide DAT dosing, resource limitations through donated DAT, low data quality with Besredka skin-sensitivity testing method unreliable in setting and underreporting of anaphylaxis | Nursing checklists, capacity building through standardized treatment protocol, physician intervention with adverse events | No | Yes | Yes |
| El-Halabi et al. | Children Immunization App (CIMA): A Non-randomized Controlled Trial Among Syrian Refugees in Zaatari Camp, Jordan | 2023 | Jordan | Non-randomized controlled trial | Infectious disease | Mobile health app implemented among 471 Syrian refugees was shown to slightly increase vaccination follow-up | 3 | Facilitators: pre-existing partnerships, pre-existing health system, low cost, cultural adaptation, alignment with political priorities  Barriers: technological failures, low health literacy, security risks | Local collaborations, health system integration, centralized coordination, information dissemination, capacity building | No | Yes | No |
| Ezard et al. | Screening and brief intervention for high-risk alcohol use in Mae La refugee camp, Thailand: A pilot project on the feasibility of training and implementation | 2010 | Thailand | Case study | Mental health (Substance use) | 1,256 males screened for high-risk alcohol use | 6 | Facilitators: Health worker availability  Barriers: Language differences | Intervention adaptation, integration in existing systems, capacity building, targeting high-risk group | No | Yes | No |
| Fetters et al. | Navigating the crisis landscape: Engaging the ministry of health and United Nations agencies to make abortion care available to Rohingya refugees | 2020 | Bangladesh | Case study | Sexual and reproductive health | 300 health workers from 37 facilities trained in sexual and reproductive health cared for 31,000 patients | 6 | Barriers: funding restrictions, negative perceptions among religious communities, varied understandings of terminology  Facilitators: leveraging pre-existing partnerships, adaptability, alignment with political priorities | Needs assessment, political support, stakeholder engagement, establishing supply chains, capacity building, partnership for referral site strengthening | No | Yes | No |
| Ghawji et al. | Feasibility, Uptake, and Results of COVID-19 Antigen Rapid  Diagnostic Tests among Refugees and Migrants in a Pilot  Project in North-West Syria | 2023 | Syria | Case study | Infectious disease | Using community health workers, 24,956 participants were anitgen tested for COVID-19 | 6 | Facilitators: designated space, positive perceptions of intervention, health worker availability, ease of access, cultural adaptability  Barriers: limited infrastructure, obtaining informed consent, cost, stigma | Piloting, local partnerships, task-shifting, health system integration, supervision, information dissemination | No | Yes | No |
| Greene et al. | Evaluation of an integrated intervention to reduce psychological distress and intimate partner violence in refugees: Results from the Nguvu cluster randomized feasibility trial | 2021 | Tanzania | Feasibility cluster randomized trial | Mental health | Among 311 Congolese women, a multi-sectoral integrated violence- and mental health-focused intervention (Nguvu) run by lay refugee incentive workers led to partial reductions in psychological stress for women experiencing intimate partner violence and psychological distress, but no decrease in violence itself. | 2 | Facilitators: Lay worker involvement, minimal training for intervention, pre-established health system, local partnerships  Barriers: Cultural differences between participants and facilitators, non standardized definition of intimate partner violence, high staff turnover | Integration into existing health systems, task shifting to lower-level providers | No | Yes | Yes |
| Greene et al. | Examining implementation of an intervention to reduce psychological distress and intimate partner violence in a refugee camp setting | 2022 | Tanzania | Cross-sectional, qualitative | Mental health | A multi-sectoral integrated violence- and mental health-focused intervention was determined to be beneficial with recommendations for adaptation through semi-structured interviews. | 6 | Facilitators: Minimal training for intervention, expert consultants, appointing local group leaders to share information about meetings  Barriers: Limited infrastructure, competing priorities, population mobility, population diversity and distribution, rainy season, scheduling issues with food distribution/etc. | Stakeholder engagement, clinical supervision, interdisciplinary approach, information dissemination | No | No | No |
| Grijalva-Eternod et al. | Evaluation of conditional cash transfers and  mHealth audio messaging in reduction of risk factors for childhood malnutrition in internally displaced persons camps in Somalia: A 2 × 2 factorial cluster-randomised controlled trial | 2023 | Somalia | 2x2 factorial cluster-randomized controlled trial | Infectious disease / Health technology | 1,430 households randomized to receive conditional cash transfers and an mHealth intervention, demonstrating that conditional cash transfers improved coverage of measles vaccine and completion of pentavalent series. mHealth improved dietary diversity but did not improve measles vaccination | 2 | Facilitators: ease of access, pre-existing partnerships, high-income institution partnership, expert consultation  Barriers: time constraints, resource limitations, weak health infrastructure, health workers shortages, mobile population, negative perceptions of intervention, low health literacy | Technology integration, information dissemination, stakeholder engagement, language adaptations, evaluation mechanisms, | No | Yes | Yes |
| Halder et al. | Understanding the challenges and gaps in community engagement interventions for COVID-19 prevention strategies in Rohingya refugees: a qualitative study with frontline workers and community representatives | 2023 | Bangladesh | Case study | Infectious disease | Risk communication and community engagement facilitates a number of good practices including community health workers, female engagement, local adaptations, and collaboration. | 6 | Facilitators: culturally appropriate interventions, preestablished health systems, pre-existing partnership  Barriers: poverty, environmental conditions, distrust of health workers, sex differences, geographic barriers, limited resources, stigma | Task-shifting, cultural adaptations, stakeholder engagement, information dissemination, needs assessment, local champions | No | No | No |
| Kaic et al | Hepatitis A control in a refugee camp by active immunization | 2001 | Croatia | Cohort implementation study | Infectious diseases | Prospective testing with immunization of seronegative individuals was more cost effective and effectively controlled hepatitis A outbreak in a Croatian refugee camp | 4 | Facilitators: sanitary surveillance of food suppliers, immunization campaign, effective vaccination  Barriers: lack of running water, overcrowding in camps, high prevalence of existing seropositive individuals | Health surveillance, testing, needs assessment | No | Yes | Yes |
| Kayali et al. | Treating Syrian refugees with diabetes and hypertension in Shatila refugee camp, Lebanon: Medecins Sans Frontieres model of care and treatment outcomes | 2019 | Lebanon | Retrospective cohort study | Noncommunicable disease | MSF model of care for diabetes and hypertension was feasible and showed significantly controlled blood sugar and pressure | 4 | Facilitators: dynamic and adapting system, resource availability  Barriers: poor infrastructure, insecurity, lack of health care coverage, poverty | Task-shifting, intervention adaptation | No | Yes | Yes |
| Keshk et al. | Offering care for victims of torture among a migrant population in a transit country: a descriptive study in a dedicated clinic from January 2017 to June 2019 | 2021 | Anonymized | Retrospective cohort study | Mental health | An MSF clinic to provide multidisciplinary care to migrants provided varied rates of mental, physiotherapy, and social care. | 6 | Facilitators: social networks, direct word of mouth advice from family, friends or community members  Barriers: cost issues (inability to pay for transportation), difficulty finding transportation, desire to focus on work | Health promotion team affiliated to clinic, regular health education sessions to decrease stigma of torture | No | Yes | No |
| Khan et al. | Implementation and Delivery of Oral Cholera Vaccination Campaigns in Humanitarian Crisis Settings among Rohingya Myanmar nationals in Cox’s Bazar, Bangladesh | 2023 | Bangladesh | Case study | Infectious disease | Seven rounds of oral cholera vaccine were implemented among 900,000 Rohingya refugees, resulting in new subsequent outbreaks. | 6 | Facilitators: international partnership, local collaboration, alignment with political priorities  Barriers: resource insufficiencies | Mobile delivery components, local champions, information dissemination, physical space, evaluation mechanisms, patient tracking and data collection, establishing supply chains | No | Yes | Yes |
| Khader et al. | Scaling the Children Immunization App (CIMA) to Support Child Refugees and Parents in the Time of the COVID-19 Pandemic: A Social Capital Approach to Scale a Smartphone Application in Zaatari Camp, Jordan | 2022 | Jordan | Qualitative cross-sectional | Health technology | CIMA was successful in disseminating risk and health information in refugee camps among 1100 children. | 6 | Facilitators: Positive receptivity from parents, quick recruitment, parent trust in medical professionals and app  Barriers: Internet connectivity and reliability, access to the clinic in winter, incompatible operating systems | Clinical and household visits, user feedback, use of local stakeholders for implementation | No | Yes | No |
| Klabbers et al. | Leveraging interactive voice response technology to mitigate COVID-19 risk in refugee settlements in Uganda: Lessons learned implementing "Dial-COVID" a toll-free mobile phone symptom surveillance and information dissemination tool | 2023 | Uganda | Case study | Health technology | Over 10,000 public health messages were disseminated to refugees regarding COVID-19 symptoms and exposures. | 6 | Facilitators: adaptability of intervention, international partnership, pre-existing collaborations  Barriers: low health literacy, COVID-19 lockdown, technological failures/connectivity issues | Intervention adaptation and language, mobile delivery components, stakeholder engagement, integration into existing health systems, capacity building, task-shifting, local champions, information dissemination, iterative support and adaptations, feedback mechanisms | No | Yes | No |
| Knust et al. | Enhancing Respiratory Disease Surveillance to Detect COVID-19 in Shelters for Displaced Persons, Thailand-Myanmar Border, 2020-2021 | 2022 | Thailand, Myanmar | Case study | Infectious disease | A surveillance guidance testing system was implemented for COVID-19, testing 6,190 specimens | 6 | Facilitators: international collaboration, pre-existing health system  Barriers: COVID-19 lockdown, resource limitations, timing constrains, language barriers, geographic barriers, non-citizen status | Community education, information dissemination, clear guidelines, centralized coordination, evaluation metrics, iterative support, health system integration | No | Yes | No |
| Koop et al. | Results of the expanded program on immunization in the Macedonian refugee camps | 2001 | North Macedonia | Case report | Infectious Disease | Starting an expanded immunization program in the absence of a stable population is problematic | 6 | Facilitators: population overall good health and lack of malnutrition  Barriers: personnel shortages, population fluctuations, security risks | International collaboration, vaccination cards, resource supplying, needs assessment | No | Yes | No |
| Korave et al | Internal displacement; an impediment to the successful implementation of planned measles supplemental activities in Nigeria, a case study of Benue State | 2021 | Nigeria | Case study | Infectious diseases | Suboptimal immunization coverage might be due to the unstable population in the IDP camps or issues with the denominator. Issues such as budgeting played a crucial row in suboptimal coverage and it is essential to maintain routine immunizations in IDP camps for children to maintain herd immunity | 6 | Facilitators: stakeholder involvement, existing framework usage  Barriers: funding issues, health worker shortage, unstable population | Post vaccine strategy identification, resource mobilization, community engagement, capacity building, feedback mechanisms, clinical supervision | No | Yes | No |
| Kouadio et al. | Outbreak of measles and rubella in refugee transit camps | 2009 | Ivory Coast | Cross-sectional | Infectious disease | Ongoing surveillance for measles and rubella through a disease surveillance team (DST) to control outbreaks. Serological testing for rubella and measles prior to immunization is critical. | 6 | Facilitators: family, community leader, and health worker involvement, to establish surveillance and mobilize resources  Barriers: administrative and logistical issues | Establishment of diseases surveillance team (DST), stakeholder engagement, interdisciplinary approaches, monitoring | No | Yes | No |
| Lam et al. | Oral Cholera Vaccine Coverage during an Outbreak and Humanitarian Crisis, Iraq, 2015 | 2017 | Iraq | Cross-sectional | Infectious disease | The experience in Iraq demonstrates that OCV campaigns can be successfully implemented as part of a comprehensive response to cholera outbreaks among high-risk populations in conflict settings | 6 | Facilitators: rapid collaboration and coordination between partners  Barriers: unavailability of vaccine, skepticism towards vaccine, absence of the decision maker at home at the time of the vaccinator’s visit, global vaccine limitations, security risks | Information dissemination,  Use of electronic tablets or data collection and GPS tracking of survey teams for immediate corrective action | No | Yes | No |
| Lawrence et al. | Trauma‐focused counseling and social effectiveness skills training interventions on impaired psychological functioning of internally displaced adolescents in Nigeria | 2020 | Nigeria | Quasi-experimental factorial design with randomized cohort trial and cluster sampling | Mental health | Trauma-focused counseling and social effectiveness skills significantly improve psychological functioning of displaced adolescents | 3 | No | No | No | Yes | Yes |
| Levy et al. | Psychological first aid through the ‘SIX Cs model’ - an intervention with migrants on the move | 2020 | Serbia | Case study | Mental health | SIX Cs method was feasible in providing mental and psychosocial support to the Presevo refugee camp | 6 | Facilitators: None  Barriers: Standardization, ethical/logical constraints, shortage of staff | Capacity building, local collaboration, local champions, information dissemination | No | No | No |
| Lyles et al. | Adoption of Electronic Medical Records for Chronic Disease Care in Kenyan Refugee Camps: Quantitative and Qualitative Prospective Evaluation | 2023 | Kenya | Case study | Health technology | An adapted mHealth app successfully reported 7,918 consultations with high completeness of reporting and satisfaction | 6 | Facilitators: intervention adaptability, positive perceptions on the intervention  Barriers: low data quality, intervention bulkiness | Integration into existing health systems, capacity building, intervention adaptation, iterative support, evaluation mechanisms | No | Yes | No |
| Mahmuda et al. | Contextual adaptation and piloting of Group Integrative Adapt Therapy (IAT-G) amongst Rohingya refugees living in Bangladesh | 2019 | Bangladesh | Case study | Mental health | Psychotherapy intervention implemented by 17 counselors among 116 clients | 6 | Facilitators: political priorities  Barriers: cultural unfamiliarity, language differences, low literacy, environmental factors, employee turnover, security concerns, stigma surrounding counseling | Evidence gathering, Stakeholder engagement, intervention adaptation, capacity building, supervision | No | Yes | No |
| Mahn et al. | Multi-level partnership to promote health services among internally displaced in eastern Burma | 2008 | Myanmar | Case study | Primary care | Health system building was successful for IDPs in Eastern Myanmar through cross border local-global partnerships, including treating 78,000 patients annually | 6 | Facilitators: health worker availability, external funding, mobile clinic infrastructure  Barriers: political resistance, insufficient supplies, security risks, lack of data collection systems | Local partners, stakeholder engagement, train-the-trainer, local autonomy, coordination strategies including patient referral, information dissemination, integrated triage approaches | No | No | No |
| McEwen et al. | Feasibility and acceptability of phone-delivered psychological therapy for refugee children and adolescents in a humanitarian setting | 2024 | Lebanon | Case study | Health technology | t-CETA delivered over the phone both created and solved logistical challenges but was ultimately considered feasible and acceptable by stakeholders. | 6 | Facilitators: strong evidence base, adaptability of intervention, familial support, flexible delivery components  Barriers: shortage of health workers, technological failures, distrust of health workers, stigma, cost | Task-shifting, intervention adaptation, familial support, mobile delivery components, capacity building, supervision, | No | No | No |
| Mellou et al | Increasing childhood vaccination coverage of the refugee and migrant population in Greece through the European programme PHILOS, April 2017 to April 2018 | 2019 | Greece | Cross-sectional | Infectious disease | Vaccination interventions led to a high vaccine coverage for the dose of the MMR vaccine including 57,615 vaccinations, but not for other vaccines. | 6 | Facilitator: NGO involvement, standardized procedures, community engagement, availability of cultural mediators  Barriers: cultural mediator barriers, camp population size changes, high turnover of population, insufficient coverage for some diseases, legal challenges, barriers to accessing healthcare services, data collection limitations | Needs assessment, NGO collaboration, database implementation, education, collaboration with political stakeholders, stakeholder engagement, healthcare center designation (physical space) | No | Yes | No |
| Mercer et al. | Psychosocial distress of Tibetans in exile: integrating western interventions with traditional beliefs and practice | 2005 | India | Cross-sectional | Mental health | Mental health services were beneficial but spiritual and cultural practices were most important in mental health care | 6 | Facilitators: cultural sensitivity, mental health awareness in the community  Barriers: training, social barriers, social stigma, language and cultural differences, sustainability | Capacity building | No | No | No |
| Metzler et al. | Improving adolescent mental health and protection in humanitarian settings: longitudinal findings from a multi‐arm randomized controlled trial of child‐friendly spaces among South Sudanese refugees in Uganda | 2023 | Uganda | Randomized controlled trial | Mental health | Both the Toolkit for Friendly Spaces in Humanitarian Settings and a standard psychological intervention were well suited as first-line mental health intervention among 1,280 adolescents | 2 | Facilitators: pre-existing partnerships, clear guidelines  Barriers: COVID-19 restrictions, language barriers, physical space closure, demotivated participants | Task-shifting, capacity building, game play, community empowerment, intervention co-design, physical space, iterative training, | No | Yes | Yes |
| Mitchell-Gillespie et al. | Sustainable support solutions for community-based rehabilitation workers in refugee camps: piloting telehealth acceptability and implementation | 2020 | Jordan | Cohort implementation study | Occupational, physical, and speech therapy / Telehealth | Telehealth can be utilized to support rehabilitation workers that serve vulnerable and marginalized populations, and in turn improve the global health status among refugee populations by reducing inequitable access to care | 6 | Facilitators: increased demand for telehealth  Barriers: Cultural beliefs, limited health worker training, faulty infrastructure, scheduling meetings on email, WiFi connection, language barrier, connectivity, language difficulties, technological illiteracy | Local champions, raising political support, capacity building through health worker training, information dissemination, needs assessment, telehealth communication through Zoom, intervention adaptation | RE-AIM and DSF | No | No |
| Momotaz Hasina et al | Implementing the Mental Health Gap Action Programme in Cox’s Bazar, Bangladesh | 2019 | Bangladesh | Cohort implementation study | Mental health | There was a positive increase in healthcare workers’ ability to provide mental health services | 6 | Facilitators: WHOs recommendation to increase capacity for mental health care, adapting mhGAP material for context of the country including translations, local organization and worker availability  Barriers: limited trained health workers, not comprehensive understanding of cultural determinants, limited documentation and health information system, referral system issues | Rapid assessment, local institute collaboration, training healthcare workers, refresher training, supervision, local collaborations, technology utilization | No | Yes | No |
| Moreau et al. | 3D technology and telemedicine in humanitarian settings | 2020 | Jordan | Case study | Surgery | Provided facial orthoses for 24 patients and limb prostheses for 29 | 6 | Facilitators: Low-cost materials  Barriers: lack of specialists, timing constraints, technological failures, return to home country, human resource cost | Multidisciplinary team, working with local partners | No | Yes | No |
| Morris et al. | Does Combining Infant Stimulation With Emergency Feeding Improve Psychosocial Outcomes for Displaced Mothers and Babies? A Controlled Evaluation From Northern Uganda | 2012 | Uganda | Randomized control trial | Maternal / child health | Among 70 participants, combining group-based psychosocial intervention with preexisting emergency feeding programs helps displaced mothers more positively interact with/care for their babies and increased maternal mood. | 2 | Facilitators: 5 sites were comparable, eventual peace accord led to more mobility  Barriers: Ethical concerns about individual randomization, distance between feeding centers and communities, security risks, environmental conditions | Local professionals as community stakeholders, physical space | No | Yes | Yes |
| Mullany et al. | The MOM Project: Delivering Maternal Health Services among Internally Displaced Populations in Eastern Burma | 2008 | Myanmar | Case report | Maternal health | Training of nearly 300 community-level providers for maternal services delivery | 6 | Facilitators: established health system  Barriers: security risks, lack of infrastructure, widely dispersed population, shortage of health workers | Train-the-trainer, iterative communication, mobile operations, health system integration, local partners, stakeholder engagement, site selection, supervision, remuneration | No | Yes | No |
| Murphy et al. | Implementation of fixed-dose combination therapy for secondary prevention of atherosclerotic cardiovascular disease among Syrian refugees in Lebanon: a qualitative evaluation | 2022 | Lebanon | Qualitative (nested) | NCD | Combination therapy was acceptable and feasible | 6 | Facilitators: Ease of medication administration, trust in medical professionals  Barriers: apprehension from unfamiliar drug, clinic closure | No | No | No | No |
| Murray et al. | An evaluation of a common elements treatment approach for youth in Somali refugee camps | 2018 | Ethiopia/Somalia | Quasi-experimental | Mental health | The CETA protocol significantly reduces post-traumatic stress symptoms and increases well-being in youth residing in refugee camps and their caregivers. | 4 | Facilitators: Relatively few sessions, common elements with an adaptable approach, local partners  Barriers: Intervention bulkiness | Local champions, intervention adaptation, piloting, integration into existing health systems | Design, Implementation, Monitoring, and Evaluation (DIME) | Yes | Yes |
| O’Connell et al. | Meeting the Sexual and Reproductive Health Needs of Internally Displaced Persons in Ethiopia's Somali Region: A Qualitative Process Evaluation | 2022 | Ethiopia | Case study | Sexual and reproductive health | Implementation of sexual and reproductive health programs among 4,500 IDPs in Ethiopia demonstrated identifiable challenges and success metrics | 6 | Facilitators: health worker availability, intervention adaptability, international collaborations, preestablished health system  Barriers: security risks, limited health worker availability, sex differences, COVID-19 restrictions, cultural and language barriers, low health literacy, environmental conditions | Local collaboration, stakeholder engagement, capacity building, intervention adaptations, integration into existing health systems, co-design of interventions, iterative adaptations | No | Yes | No |
| O’Laughlin et al. | A cohort study to assess a communication intervention to improve linkage to HIV care in Nakivale Refugee Settlement, Uganda | 2021 | Uganda | Cohort implementation study | Health technology | For those who do not receive linkage to care on the day of diagnosis, a phone call/SMS intervention to encourage linkage is effective. | 4 | Facilitators: Linkage on the day of diagnosis    Barriers: No access to mobile phone, low or no literacy | No | No | Yes | No |
| O’Laughlin et al. | Clinic-based routine voluntary HIV testing in a refugee settlement in Uganda | 2014 | Uganda | Cohort implementation study | Infectious Disease | Clinic-based testing in a refugee settlement was feasible and resulted in 6-fold HIV identification weekly | 3 | No | No | No | Yes | No |
| Oladeji et al | Integrating immunisation services into nutrition sites to improve immunization status of internally displaced persons’ children living in Bentiu protection of civilian state, South Sudan | 2019 | South Sudan | Cohort implementation study | Infectious disease | Integrating immunization systems within the healthcare delivery program increased the number of immunized children and the dropout rate in vaccination was significantly lower in the outpatient therapeutic program (OTP) centers than the primary health care centers (PHC) | 4 | Facilitators: funding, nutrition services as incentive, health education, community nutrition volunteers, tracking default vaccination sessions  Barriers: time constraints of caregivers, absences at vaccination clinics, security risks, forgetting vaccination cards and having to reschedule immunizations | Collaborative teams, health education, defaulter tracking, integration of services within existing infrastructure, stakeholder approval | Global vaccine action plan | Yes | No |
| Ope et al. | Evaluation of the Field Performance of ImmunoCard STAT! R Rapid Diagnostic Test for Rotavirus in Dadaab Refugee Camp and at the Kenya-Somalia Border | 2017 | Kenya | Field function study | Infectious disease | Diagnosing rotavirus in refugee camps is made more feasible through an RDT called ICS-RV, which ultimately fulfills the WHO ASSURED criteria for point-of-care testing. | 6 | Facilitators: Affordable, minimal training for intervention, minimal resource requirements  Barriers: Potential presence of other pathogens, different stages of illness at intervention, uncontrolled room temp during incubation | Clinical supervision | No | Yes | No |
| Phares et al. | Mass vaccination with a two-dose oral cholera vaccine in a long-standing refugee camp, Thailand | 2016 | Thailand | Cohort implementation study | Infectious disease | 63,057 oral cholera vaccine doses to 35,399 refugees with relatively low adverse effects | 4 | No | Information dissemination, stakeholder engagement, evidence gathering, mobile and frequently available vaccine teams | No | Yes | Yes |
| Polonsky et al. | Epidemiological, clinical, and public health response characteristics of a large outbreak of diphtheria among the Rohingya population in Cox's Bazar, Bangladesh, 2017 to 2019: A retrospective study | 2021 | Bangladesh | Retrospective review | Infectious disease | 7.064 cases of diphtheria with 89% of eligible patients being subsequently vaccinated | 4 | No | Case isolation and treatment, contact tracing | No | Yes | No |
| Porta et al. | Feasibility of a preventive mass vaccination campaign with two doses of oral cholera vaccine during a humanitarian emergency in South Sudan | 2014 | South Sudan | Cohort implementation study | Infectious disease | Assessing feasibility and acceptability of a mass vaccine campaign using 2 strategies showed high coverage capabilities | 4 | Facilitators: High vaccination acceptance, high thermostability of Shanchol, health worker availability  Barriers: Vaccination card matching for tracking pt doses, cold chain ruptures, heat exposure, resource intensive strategies, storage volume, multiple dosage requirement | Stakeholder engagement, social mobilization, feedback mechanisms, capacity building, task shifting | No | Yes | No |
| Purdin et al. | Reducing maternal mortality among Afghan refugees in Pakistan | 2009 | Pakistan | Cohort implementation study | Maternal health | Emergency obstetric care centers improved maternal mortality, prenatal coverage, postnatal coverage, and number of births attended by a skilled attendant | 4 | Facilitators: presence of trained health workers and community health workers, integration into existing system, pre-established feedback mechanisms  Barriers: social restrictions for women, cultural norms limiting travel, security risks | Capacity building, community education, centralized coordination, technology integration, collaborating with local partners, iterative training | No | Yes | Yes |
| Quosh | Comprehensive mental health and  psychosocial support case  management and indicative  care pathways within  humanitarian settings | 2016 | Syria | Case study | Mental health | Case management can support uptake of mental health services, with this care program managing 6,000 clients | 6 | Facilitators: Health worker availability, political priorities  Barriers: low resources | Needs assessment, referral pathways, local champions, political integration, integration into existing health systems | No | Yes | No |
| Rabiou et al. | Implementation and contribution of temperature screening and handwashing practice at points of entry for COVID-19 pandemic response in a humanitarian crisis setting | 2022 | Niger | Case study | Infectious disease | A COVID-19 surveillance program successfully screened 169,475 migrants for fever and high compliance with hand washing regulations | 6 | Facilitators: clear guidelines, culturally appropriate intervention  Barriers: none | Task-shifting, capacity building, cultural adaptation, information dissemination, resource supply, supervision | No | Yes | No |
| Rah et al. | Program experience with micronutrient powders and current evidence | 2012 | Bangladesh, Nepal, Kenya | Systematic review | Nutrition | Self-reported acceptability of micronutrient powders and adherence were variable (Nepal > Bangladesh > Kenya) | 4 | Facilitators: internationally validated program  Barriers: concerns about endemic diseases like malaria, insufficient social marketing, inadequate staff training | Stakeholder engagement, government permission, resource mobilization, capacity building, packaging design, monitoring and evaluation | No | Yes | Yes |
| Rainey et al. | Rapid monitoring in vaccination campaigns during emergencies: the post-earthquake campaign in Haiti | 2013 | Haiti | Cohort implementation study | Infectious disease | Rapid monitoring of vaccination campaigns is only marginally beneficial in achieving immunization targets in temporary camps | 4 | Facilitators: government support  Barriers: immigration/emigration, large number of camps, small number of vaccinations, participation | Rapid monitoring, standardized triage approach | No | Yes | No |
| Ravicz et al. | Using Intervention Mapping methodology to design an HIV linkage intervention in a refugee settlement in rural Uganda | 2022 | Uganda | Qualitative cross-sectional | Infectious Disease | Stakeholders and community members designed a feasible intervention to promote linkage to HIV care based on a detailed discussion of the causes and consequences of untreated HIV. | 6 | Facilitators: participation from diverse stakeholders  Barriers: frequent change to guidelines, rapid staff and leadership turnover | Intervention mapping, capacity building, stakeholder engagement | No | No | No |
| Rijken et al. | Obstetric ultrasound scanning by local health workers in a refugee camp on the Thai-Burmese border | 2009 | Thailand | Qualitative cross-sectional | Maternal and child health | Locally trained health workers in a refugee camp on the Thai-Burmese border obtained accurate fetal biometry measurements with obstetric ultrasound for gestational age among 349 patients | 6 | Facilitators: trained health workers, robust ultrasound machines  Barriers: ultrasound instrument cost, specialist time constraints | Capacity building, task-shifting | No | Yes | No |
| Rossi et al | Design and implementation of a hospital information system for the Palestine Red Crescent Society in Lebanon | 2009 | Lebanon | Case study | Health technology | Improved care/tracking via implementing a case-mix hospital information system for Palestinian refugees in Lebanon at Palestine Red Crescent Society hospitals. | 6 | Facilitators: Health worker availability, pre-established health system, pre-existing partnerships between clerks and hospital administration  Barriers: Intervention complexity and bulkiness requiring expansion, funding restrictions | Stakeholder engagement, capacity building, needs assessment, semi-clinical supervision (data managers) | No | No | No |
| Rutta et al. | Prevention of mother-to-child transmission of HIV in a refugee camp setting in Tanzania | 2008 | Tanzania | Cohort implementation study | Infectious disease | PMTCT (prevention of mother to child HIV transmission) program was successfully integrated into existing antenatal care services and was acceptable to majority of pregnant women | 4 | Facilitators: Community participation and male involvement, collaboration with government  Barriers: Misconceptions of medication, loss to follow up, lack of cross border cooperation | Capacity building, piloting program, involvement of community members, collaboration with other programs | No | Yes | No |
| Saeed Khan et al. | Mental health considerations of a humanitarian crisis: Identification of needs and delivery of services to Afghan child and adolescent refugees in Qatar | 2022 | Qatar | Case study | Mental health | Successful referral of 30 individuals to mental health services | 6 | Facilitators: none  Barriers: security risks | Stakeholder involvement, interdisciplinary approach, local champion | No | Yes | No |
| Sami et al. | An analytic perspective of a mixed methods study during humanitarian crises in South Sudan: translating facility- and community-based newborn guidelines into practice | 2021 | South Sudan | Case study | Child health | Newborn health interventions are acceptable and feasible after two-day simulation and provision of supplies; knowledge did not necessarily lead to adoption of interventions in the community. | 6 | Facilitators: Provision of supplies and training  Barriers: Mass displacement, security risks, high staff turnover, cost, competing health priorities | Involvement of community health workers | No | No | No |
| Sami et al. | "You have to take action": changing knowledge and attitudes towards newborn care practices during crisis in South Sudan | 2017 | South Sudan | Mixed methods pre/post descriptive study | Child health | Conducting training has the potential to increase health workers' knowledge on neonatal health post-training | 6 | Facilitators: health care workers positive attitudes  Barriers: Lack of newborn-specific protocols, resources, previously inaccurate practices, cultural norms opposing clinical practices | Information dissemination through written materials, capacity building | No | Yes | No |
| Sampson et al. | Addressing barriers to accessing family planning  services using mobile technology intervention  among internally displaced persons in Abuja,  Nigeria | 2023 | Nigeria | Case study | Health technology | The Linking Underserved Populations to Sexual and Reproductive Health Services intervention increased awareness of family planning, spousal consent, and contraceptive prevalence rate among 103 IDPs | 4 | Facilitators: cultural appropriateness, familial support, pre-existing health system, available infrastructure  Barriers: security risks, environmental conditions, male consent, low health literacy, negative perceptions towards the intervention, mobile population | Integration into existing health systems, community education, game play, local champions, stakeholder engagement, local empowerment | No | Yes | Yes |
| Sarker et al. | Effective maternal, newborn and child health programming among Rohingya refugees in Cox's Bazar, Bangladesh: Implementation challenges and potential solutions | 2020 | Bangladesh | Qualitative | Maternal / child health | CFIR should be supplemented with context and security domains for use in humanitarian contexts | 6 | Facilitators: motivating health workers, task shifting for community health workers, capacity building emergency obstetric care  Barriers: high turnover and poor staff retention, overlapping services, weak referrals, complex health systems, security risks | No | CFIR | No | No |
| Seal et al. | Use of an adapted participatory learning and action cycle to increase knowledge and uptake of child vaccination in internally displaced persons camps (IVACS): A cluster-randomised controlled trial | 2023 | Somalia | Randomized controlled trial | Infectious disease | An adapted Participatory Learning and Action program improved caregiver knowledge, coverage of measles vaccine, and completion of pentavalent vaccine across approximately 1,520 IDPs | 2 | Facilitators: social interconnectedness, positive perceptions towards the intervention, re-established health system, pre-existing partnerships, international partnerships  Barriers: security risks, environmental conditions, geographic barriers, resource insufficiencies, low health literacy, time constraints | Local partnerships, local champions, task-shifting, intervention adaptation, capacity building, iterative support, stakeholder engagement, group activity, information dissemination, mobile health delivery components, feedback | No | Yes | Yes |
| Sechriest et al. | Orthopaedic care aboard the USNS Mercy during Operation Unified Assistance after the 2004 Asian tsunami. A case series | 2008 | Southeast Asia | Case study | Orthopedics | Navy and Project HOPE orthopedic surgeons through the USNS Mercy met the need for complex orthopedic care and complemented local medical teams | 6 | Facilitators: Consultation to US experts  Barriers limited time allotted for the mission in each location which required accelerated discharge, language barriers, storms | Mobile delivery components, specialized physical space, establishing supply chains | No | Yes | No |
| Sethi et al. | Community-Based Noncommunicable Disease (NCD) Care for Syrian Refugees in Lebanon | 2017 | Lebanon | Cohort implementation study | NCD | 500 refugee outreach volunteers cared for 387 patients with NCDs | 4 | No | Mobile clinics, needs assessment, raising political support, group facilitation, technology integration | No | Yes | No |
| Shaikh | Nurses' use of global information systems for provision of outreach reproductive health services to internally displaced persons | 2008 | Somalia | Case study | Health technology | Use of a global information systems (GIS) map was used to facilitated 3,095 mobile outreach clinic visits to IDPs in the outskirts of Baidoa City, Somalia | 6 | Facilitators: local non-governmental organization, funding from United Nations Population Fund  Barriers: travel in rain-affected passages, security risks | Integration to healthcare systems, identification of strategic areas for services | No | Yes | No |
| Sheikh et al. | Combined use of inactivated and oral poliovirus vaccines in refugee camps and surrounding communities - Kenya, December 2013 | 2014 | Kenya | Cohort implementation study | Infectious disease | Polio vaccine distribution to 2,000 children by 299 teams | 4 | Facilitators: Alignment with political priorities, adaptability, mobile sites  Barriers: Cost, operational complexity, caregiver mistrust, limited trained health workers | Local champions, needs assessment, information dissemination, checklists, mobile delivery sites | No | Yes | No |
| Shortall et al. | On the ferries: The unmet health care needs of transiting refugees in Greece | 2017 | Greece | Retrospective review | Primary care | An integrated, flexible and multidisciplinary approach to respond to needs of a transiting population like the Refugee Ferry Project responds to the population’s needs by continuous reassessment, mobilization capacity, and adapting to evolving situations | 6 | Facilitators: intervention flexibility  Barriers: political agreements to deport migrants, physical space, time, limited health workers, language barriers, differing belief systems, and resource limitations | Physical space,  partnership with ferry operator, information dissemination | No | Yes | No |
| Sibai et al | Lessons learned in the provision NCD primary care to Syrian refugee and host communities in Lebanon: the need to ‘act locally and think globally” | 2020 | Lebanon | Cohort implementation study | Non communicable diseases | Successful implementation to screen 1876 patients for NCDs with high satisfaction but barriers to scaling | 3 | Facilitators: Use of clear WHO interventions, healthcare worker training, local and well-equipped health facilities  Barriers: resource limitations, space constraints on mobile health units, harsh weather, low literacy among health workers, time constraint | Collaboration with local and international organizations, needs assessment, targeting implementation sites, capacity building, feedback mechanism | Precede-Proceed | Yes | No |
| Sonderegger et al. | Trauma rehabilitation for war-affected persons in northern Uganda: A pilot evaluation of the EMPOWER programme | 2011 | Uganda | Cohort non-randomized control trial | Mental health | EMPOWER CBT programme significantly reduced depression and anxiety symptoms among internally displaced persons | 4 | No | Intervention adaptation, local partners, information dissemination, game play | No | Yes | Yes |
| Stein et al. | Cash, COVID-19 and aid cuts: a mixed-method impact evaluation among South Sudanese refugees registered in Kiryandongo settlement, Uganda | 2022 | Uganda | Quasi-experimental mixed methods | Infectious disease | Households receiving cash transfers were more food secure, with psychological wellbeing, and more likely to seek care in private facilities. Cash transfer had no impact on preventative measures against COVID-19. | 3 | Facilitators: Established resource supply chain with earlier administration of aid  Barriers: Additional food needs, insufficient land to grow food, cuts in aid from other sources | No | No | Yes | Yes |
| Sullivan et al. | Using simple acupressure and breathing techniques to improve mood, sleep and pain management in refugees: a peer-to-peer approach in a Rohingya refugee camp | 2019 | Bangladesh | Cross Sectional | Mental health | Acupressure and mindful breathing techniques, taught peer-to-peer via trusted Rohingya CHWs, improved mental health complaints, empowered refugees to use self-care, and promoted caring relationships within families. | 6 | Facilitators: engagement among Rohingya CHWs, pre-existing pictures and demonstration teaching methods  Barriers: embarrassment at practicing new physical techniques, sex disparities with females less vocal | Train-the-trainer, task-shifting, low-cost tools, peer-to-peer teaching | No | Yes | No |
| Talley et al. | Challenges to the programmatic implementation of ready to use infant formula in the post-earthquake response, Haiti, 2010: a program review | 2013 | Haiti | Retrospective review | Child health | Baby tents provided formula to 590 infants | 6 | Facilitators: NGO support in enrolling patients  Barriers: Difficult to access/overcrowded hospitals, inaccurate equipment, low data quality | Local collaboration, physical space establishment | No | Yes | No |
| Tarannum et al. | Integrating mental health into primary health care in Rohingya refugee settings in Bangladesh: Experiences of UNHCR | 2019 | Bangladesh | Case study | Mental health | Implementation of the mhGAP program resulted in 1,200 clinical mental health consultations within 9 months. Integrating mental health into primary health care is feasible | 6 | Facilitators: use of existing verified program, trained health workers  Barriers: limited resources, staff turnover, language barriers, stigma, limited capacity for family-focused mental health support, limited mental health facilities, trouble applying trained knowledge | Stakeholder engagement, needs assessment, capacity building, information dissemination, clinical supervision, monitoring and feedback | No | Yes | No |
| Tol et al | Guided self-help to reduce psychological distress in South Sudanese female refugees in Uganda: a cluster randomized trial | 2020 | Uganda | Cluster randomized control trial | Mental health | Among 613 participants, guided self-help intervention reduced psychological distress. The intervention did not vary with trauma exposure, length of time in settlement or baseline level of distress. | 2 | Facilitators: Minimal resources required for training and supervision  Barriers: limited resources, environmental stressors, poor data quality, low literacy | Engaging local stakeholders, needs assessment, facilitator training, safety measures, capacity building, information dissemination | No | Yes | Yes |
| Van Boetzelaer et al. | Evaluation of community based surveillance in the Rohingya refugee camps in Cox's Bazar, Bangladesh, 2019 | 2020 | Bangladesh | Case study | Infectious disease | 97,340 households were included in a community-based surveillance program to identify early epidemic diseases, which covered over 85% of the population. | 6 | Facilitators: health worker availability, clear guidelines, intentional interprofessional recruitment  Barriers: time constraints, intervention complexity, poor data quality, duplicated services | Task-shifting, capacity building, accurate data monitoring, supervision | No | Yes | No |
| Varkey et al. | Measles vaccination response during Kosi floods, Bihar, India 2008 | 2009 | India | Case study | Infectious disease | A measles vaccination campaign for children 6 months-14 years old prevented large scale measles outbreaks and death in camps. | 6 | Facilitators: political priorities and support, support from civil organizations  Barriers: large-scale destruction of the road network by floods, lack of mobile phones, already established surveillance systems to control outbreaks | Supervision, mobility arrangement, cold chain maintenance, local champions, information dissemination, available means of transport, centralized coordination | No | Yes | No |
| Vincent et al. | Simple spectacles for adult refugees on the Thailand-Burma border | 2006 | Thailand-Burma border | Retrospective cohort | Ophthalmology | Among 7,219 clinical visits, training refugee health workers has allowed for sustainable, low-cost spectacle provision to a large population over an extensive geographic area in a challenging environment. | 6 | Facilitators: low cost, access to the camps, improved security, and well-coordinated health system  Barriers: dynamic, volatile atmosphere | Capacity building, centralized coordination, community education, establishing physical infrastructure, local champions | No | Yes | No |
| Von Roenne et al. | Reproductive health services for refugees by refugees: an example from Guinea | 2010 | Guinea | Case study | Sexual and reproductive health | Reproductive health services can be planned and implemented by refugees for refugees with sustained funding and assistance | 6 | Facilitators: community-based counseling, training community members  Barriers: funds, trust within leadership | Multidisciplinary collaboration, local champions | No | Yes | No |
| Warren et al. | Systematic review of the evidence on the effectiveness of sexual and reproductive health interventions in humanitarian crises | 2015 | LMICs | Systematic review | Sexual and reproductive health | Several evidence-based SRH interventions may be effective for young people in humanitarian and LMIC settings | 5 | Facilitators: No  Barriers: exclusion criteria of SRH intervention in patient population less than 10 y/o | No | No | Yes | No |
| White et al. | Could the supertowel be used as an alternative hand cleaning product for emergencies? An acceptability and feasibility study in a refugee camp in Ethiopia | 2019 | Ethiopia | Cross-sectional | Infectious disease | The supertowel could be acceptable and useful for hand cleaning as a complement to soap use. | 6 | Facilitators: Positive perceptions towards the intervention  Barriers: Environmental conditions; adaptation time for new behaviors | Information dissemination | No | No | No |
| Wilton et al. | Adapting Reach up and Learn in Crisis and Conflict Settings: An Exploratory Multiple Case Study | 2023 | Bangladesh, Syria, Venezuela | Case study | Health technology | Reach Up and Learn coupled with interactive voice responses, rapid adaptations, and lactation counseling improved responsiveness to needs of young children, addressed family needs, and led to high satisfaction among 2400 households in Bangladesh, Syria, and Venezuela, respectively | 6 | Facilitators: international partnerships, site similarity, familial support, trust of health workers, originally designed for scale  Barriers: COVID-19 restrictions, security risks, low health literacy, low data quality, resource insufficiencies, poverty, time constraints | Cultural adaptation of intervention, integration into existing health systems, needs assessment, local collaboration, game play, iterative adaptation, mobile delivery components, stakeholder engagement, intervention co-design | No | Yes | No |
