# Supplementary material for: Innovative strategies and implementation science approaches for health delivery among migrants in humanitarian settings: A scoping review
Source: PLOS Glob Public Health. 2024 Dec 2;4(12):e0003514. doi: 10.1371/journal.pgph.0003514 (PMC11611092; doi:10.1371/journal.pgph.0003514)
Supplement: S2 Appendix — This file provides a complete list of studies included in the scoping review, in order of appearance of Table 1. (DOCX) [file pgph.0003514.s002.docx]

S2 Appendix: References for studies included in the scoping review presented in order as they appear in Table 1

1. Abdullahi SA, Smelyanskaya M, John S, et al. Providing TB and HIV outreach services to internally displaced populations in Northeast Nigeria: Results of a controlled intervention study. PLoS Med. 2020 Sep 1;17(9):e1003218.
2. Ali Y, Ahmed A, Siddig EE, et al. The role of integrated programs in the prevention of COVID-19 in a humanitarian setting. Trans R Soc Trop Med Hyg. 2022 Mar 1;116(3):193–6.
3. Amani A, Bita A, Nkwele IM, et al. Preventive mass vaccination campaign against meningococcal meningitis in refugee camps of Cameroon during the COVID-19 pandemic: vaccination coverage, challenges, best practices and lessons learned. International Journal of Tropical Medicine. 2021;41–6.
4. Beshr IA, Beshr MS, Al-Qubati HA. Polio outbreak response, Yemen. Bull World Health Organ. 2023 Dec 1;101(12):808-812. doi: 10.2471/BLT.23.290122.
5. Centers for Disease Control and Prevention (CDC). Emergency measles control activities--Darfur, Sudan, 2004. MMWR Morb Mortal Wkly Rep. 2004 Oct 1;53(38):897-9.
6. Chowdhury AT, Kundu S, Sultana ZZ, Hijazi HHA, Hossain A. A formative research to explore the programmatic approach of vaccinating the Rohingya refugees and host communities against COVID-19 infection in Bangladesh. BMC Health Serv Res. 2023 Aug 31;23(1):937. doi: 10.1186/s12913-023-09945-z.
7. Coldiron ME, Lasry E, Bouhenia M, et al. Intermittent preventive treatment for malaria among children in a refugee camp in Northern Uganda: lessons learned. Malar J. 2017 May 23;16(1):1–8.
8. Donkeng-Donfack VF, Tchatchueng-Mbougua JB, Abanda NN, et al. A cost-benefit algorithm for rapid diagnosis of tuberculosis and rifampicin resistance detection during mass screening campaigns. BMC Infect Dis. 2022 Mar 4;22(1):219.
9. Eisenberg N, Panunzi I, Wolz A, et al. Diphtheria Antitoxin Administration, Outcomes, and Safety: Response to a Diphtheria Outbreak in Cox's Bazar, Bangladesh. Clin Infect Dis. 2021 Oct 5;73(7):e1713-e1718.
10. Ghawji H, AlYousfi MN, Satyanarayana S, Wilson N, Tomeh L, Alkhellov H, et al. Feasibility, Uptake, and Results of COVID-19 Antigen Rapid Diagnostic Tests among Refugees and Migrants in a Pilot Project in North-West Syria. Trop Med Infect Dis. 2023 May 16;8(5):281. doi: 10.3390/tropicalmed8050281.
11. Grijalva-Eternod CS, Jelle M, Mohamed H, Waller K, Osman Hussein B, Barasa E, et al. Evaluation of conditional cash transfers and mHealth audio messaging in reduction of risk factors for childhood malnutrition in internally displaced persons camps in Somalia: A 2 × 2 factorial cluster-randomised controlled trial. PLoS Med. 2023 Feb 27;20(2):e1004180. doi: 10.1371/journal.pmed.1004180.
12. Halder CE, Hasan MA, Mohamud YM, Nyawara M, Okello JC, Mizan MN, et al. Understanding the challenges and gaps in community engagement interventions for COVID-19 prevention strategies in Rohingya refugees: a qualitative study with frontline workers and community representatives. Front Public Health. 2023 Aug 3;11:1169050. doi: 10.3389/fpubh.2023.1169050.
13. Kaic B, Borcic B, Ljubicic M, et al. Hepatitis A control in a refugee camp by active immunization. Vaccine. 2001 Jun 14;19(27):3615–9.
14. Khan AI, Islam MT, Khan ZH, Tanvir NA, Amin MA, Khan II, et al. Implementation and Delivery of Oral Cholera Vaccination Campaigns in Humanitarian Crisis Settings among Rohingya Myanmar nationals in Cox's Bazar, Bangladesh. Vaccines (Basel). 2023 Apr 14;11(4):843. doi: 10.3390/vaccines11040843.
15. Knust B, Wongjindanon N, Moe AA, Herath L, Kaloy W, Soe TT, et al. Enhancing Respiratory Disease Surveillance to Detect COVID-19 in Shelters for Displaced Persons, Thailand-Myanmar Border, 2020-2021. Emerg Infect Dis. 2022 Dec;28(13):S17-S25. doi: 10.3201/eid2813.220324.
16. Koop DG, Jackson BM, Nestel G. Results of the expanded program on immunization in the Macedonian refugee camps. Am J Public Health. 2001;91(10):1656–9.
17. Korave J, Bawa S, Ageda B, et al. Internal displacement; an impediment to the successful implementation of planned measles supplemental activities in Nigeria, a case study of Benue State. Vaccine. 2021 Nov 17;39 Suppl 3:C76–81.
18. Kouadio IK, Koffi AK, Attoh-Toure H, et al. Outbreak of measles and rubella in refugee transit camps. Epidemiol Infect. 2009 Nov;137(11):1593–601.
19. Lam E, Al-Tamimi W, Russell SP, et al. Oral Cholera Vaccine Coverage during an Outbreak and Humanitarian Crisis, Iraq, 2015. Emerg Infect Dis. 2017 Jan 1;23(1):38.
20. Mellou K, Silvestros C, Saranti-Papasaranti E, et al. Increasing childhood vaccination coverage of the refugee and migrant population in Greece through the European programme PHILOS, April 2017 to April 2018. Eurosurveillance. 2019 Jul 7;24(27):1.
21. O’Laughlin KN, Kasozi J, Walensky RP, et al. Clinic-based routine voluntary HIV testing in a refugee settlement in Uganda. J Acquir Immune Defic Syndr. 2014 Dec 12;67(4):409.
22. Oladeji O, Campbell P, Jaiswal C, et al. Integrating immunisation services into nutrition sites to improve immunisation status of internally displaced persons' children living in Bentiu prot65ection of civilian site, South Sudan. Pan Afr Med J. 2019 Jan 16;32:28.
23. Ope M, Nyoka R, Unshur A, et al. Evaluation of the Field Performance of ImmunoCard STAT!® Rapid Diagnostic Test for Rotavirus in Dadaab Refugee Camp and at the Kenya–Somalia Border. Am J Trop Med Hyg. 2017 Jun 6;96(6):1302.
24. Phares CR, Date K, Travers P, et al. Mass vaccination with a two-dose oral cholera vaccine in a long-standing refugee camp, Thailand. Vaccine. 2016 Jan 2;34(1):128-33.
25. Polonsky JA, Ivey M, Anam Mazhar MK, et al. Epidemiological, clinical, and public health response characteristics of a large outbreak of diphtheria among the Rohingya population in Cox’s Bazar, Bangladesh, 2017 to 2019: A retrospective study. PLoS Med. 2021 Apr 1;18(4):e1003587.
26. Porta MI, Lenglet A, de Weerdt S, et al. Feasibility of a preventive mass vaccination campaign with two doses of oral cholera vaccine during a humanitarian emergency in South Sudan. Trans R Soc Trop Med Hyg. 2014 Dec;108(12):810-5.
27. Rabiou LM, Oumarou B, Anya BM, Kaya MS, Didier T, Nsiari-Muzeyi BJ, et al. Implementation and contribution of temperature screening and handwashing practice at points of entry for COVID-19 pandemic response in a humanitarian crisis setting. Pan Afr Med J. 2022 Nov 7;43:127. doi:10.11604/pamj.2022.43.127.28171.
28. Rainey JJ, Sugerman D, Brennan M, et al. Rapid monitoring in vaccination campaigns during emergencies: the post-earthquake campaign in Haiti. Bull World Health Organ. 2013 Dec 12;91(12):957.
29. Ravicz M, Muhongayire B, Kamagaju S, et al. Using Intervention Mapping methodology to design an HIV linkage intervention in a refugee settlement in rural Uganda. AIDS Care. 2022 Apr;34(4):446-458.
30. Rutta E, Gongo R, Mwansasu A, et al. Prevention of mother-to-child transmission of HIV in a refugee camp setting in Tanzania. Glob Public Health. 2008;3(1):62-76.
31. Seal AJ, Mohamed HA, Stokes-Walter R, Mohamed S, Abdille AM, Yakowenko E, et al. Use of an adapted participatory learning and action cycle to increase knowledge and uptake of child vaccination in internally displaced persons camps (IVACS): A cluster-randomised controlled trial. Vaccine. 2023 May 5;41(19):3038-3046. doi:10.1016/j.vaccine.2023.02.016.
32. Sheikh MA, Makokha F, Hussein AM, et al. Combined use of inactivated and oral poliovirus vaccines in refugee camps and surrounding communities - Kenya, December 2013. MMWR Morb Mortal Wkly Rep. 2014 Mar 21;63(11):237-41.
33. Stein D, Bergemann R, Lanthorn H, et al. Cash, COVID-19 and aid cuts: a mixed-method impact evaluation among South Sudanese refugees registered in Kiryandongo settlement, Uganda. BMJ Glob Health. 2022 May 1;7(5):e007747.
34. Van Boetzelaer E, Chowdhury S, Etsay B, Faruque A, Lenglet A, Kuehne A, et al. Evaluation of community based surveillance in the Rohingya refugee camps in Cox's Bazar, Bangladesh, 2019. PLoS One. 2020 Dec 23;15(12):e0244214. doi:10.1371/journal.pone.0244214.
35. Varkey S, Krishna G, Pradhan N, et al. Measles vaccination response during Kosi floods, Bihar, India 2008. Indian Pediatr. 2009 Nov;46(11):997–1002.
36. White S, Petz JF, Desta K, et al. Could the Supertowel be used as an alternative hand cleaning product for emergencies? An acceptability and feasibility study in a refugee camp in Ethiopia. PLoS One. 2019 May 7;14(5):e0216237.
37. Akhtar A, Giardinelli L, Bawaneh A, Awwad M, Al-Hayek H, Whitney C, Jordans MJD, Sijbrandij M, Cuijpers P, Dawson K, Bryant R. Feasibility trial of a scalable transdiagnostic group psychological intervention for Syrians residing in a refugee camp. Eur J Psychotraumatol. 2021 Jun 30;12(1):1932295.
38. Bastin P, Bastard M, Rossel L, Melgar P, Jones A, Antierens A. Description and predictive factors of individual outcomes in a refugee camp based mental health intervention (Beirut, Lebanon). PLoS One. 2013;8(1):e54107.
39. Blackwell MA, Yeater EA, Ahmad S, et al. Sustaining Psychotherapist Effectiveness and Independence: An Exploratory Study With Displaced Persons in Kurdistan, Iraq. Traumatology. 2022 Sep 29. Available from: <https://static1.squarespace.com/static/6491e1ccf0fb1073e07d83dd/t/64936c3226a07876708d4fe7/1687383091379/Traumatology+Jiyan.pdf>
40. Bolton P, Bass J, Betancourt T, et al. Interventions for depression symptoms among adolescent survivors of war and displacement in northern Uganda: a randomized controlled trial. JAMA. 2007 Aug 1;298(5):519-27.
41. Borja A, Khondaker R, Durant J, et al. Child-centred, cross-sectoral mental health and psychosocial support interventions in the Rohingya response: A field report by Save the Children. Intervention. 2019 May 1;17(2):231–7.
42. Bosqui T, McEwen FS, Chehade N, Moghames P, Skavenski S, Murray L, et al. What drives change in children receiving telephone-delivered Common Elements Treatment Approach (t-CETA)? A multiple n = 1 study with Syrian refugee children and adolescents in Lebanon. Child Abuse Negl. 2023 Aug 21:106388. doi:10.1016/j.chiabu.2023.106388.
43. Bryant RA, Bawaneh A, Awwad M, et al. Effectiveness of a brief group behavioral intervention for common mental disorders in Syrian refugees in Jordan: A randomized controlled trial. PLoS Med. 2022 Mar 17;19(3):e1003949.
44. Bryant RA, Malik A, Aqel IS, et al. Effectiveness of a brief group behavioural intervention on psychological distress in young adolescent Syrian refugees: A randomised controlled trial. PLoS Med. 2022 Aug 12;19(8):e1004046.
45. Bryant RA, Bawaneh A, Awwad M, Al-Hayek H, Giardinelli L, Whitney C, et al. Twelve-month follow-up of a randomised clinical trial of a brief group psychological intervention for common mental disorders in Syrian refugees in Jordan. Epidemiol Psychiatr Sci. 2022 Nov 15;31:e81. doi:10.1017/S2045796022000658.
46. Cohen F, Yaeger L. Task-shifting for refugee mental health and psychosocial support: A scoping review of services in humanitarian settings through the lens of RE-AIM. Implement Res Pract. 2021 Mar 17;2:2633489521998790.
47. Corna F, Tofail F, Chowdhury MR, et al. Supporting maternal mental health of Rohingya refugee women during the perinatal period to promote child health and wellbeing: A field study in Cox’s Bazar. Intervention. 2019 May 1;17(2):160–8.
48. Crombach A, Siehl S. Impact and cultural acceptance of the Narrative Exposure Therapy in the aftermath of a natural disaster in Burundi. BMC Psychiatry. 2018 Jul 18;18(1).
49. Dyer GM, Biswas M. Psychological and psychiatric care for Rohingya refugees in Bangladesh. Intervention. 2019;17(2)217.
50. Ezard N, Debakre A, Catillon R. Screening and brief intervention for high-risk alcohol use in Mae La refugee camp, Thailand: a pilot project on the feasibility of training and implementation. Intervention. 2010 Nov;8(3):223–32.
51. Greene MC, Scognamiglio T, Likindikoki SL, et al. Examining implementation of an intervention to reduce psychological distress and intimate partner violence in a refugee camp setting. Glob Public Health. 2022 Nov;17(11):2868-2882.
52. Greene MC, Likindikoki S, Rees S, et al. Evaluation of an integrated intervention to reduce psychological distress and intimate partner violence in refugees: Results from the Nguvu cluster randomized feasibility trial. PLoS One. 2021 Jun 18;16(6):e0252982.
53. Keshk M, Harrison R, Kizito W, et al. Offering care for victims of torture among a migrant population in a transit country: a descriptive study in a dedicated clinic from January 2017 to June 2019. Int Health. 2021 Mar 1;13(2):89–97.
54. Lawrence KC, Falaye AO. Trauma-focused counselling and social effectiveness skills training interventions on impaired psychological functioning of internally displaced adolescents in Nigeria. J Community Appl Soc Psychol. 2020 Nov 1;30(6):616–27.
55. Levy E, Farchi M, Gidron Y, et al. Psychological first aid through the ‘SIX Cs model’ − an intervention with migrants on the move. Intervention. 2020 May 1;18(1):71–7.
56. Mahmuda M, Miah MA, Elshazly M, et al. Contextual adaptation and piloting of Group Integrative Adapt Therapy (IAT-G) amongst Rohingya refugees living in Bangladesh. Intervention. 2019 May 1;17(2):149–59.
57. Mercer SW, Ager A, Ruwanpura E. Psychosocial distress of Tibetans in exile: Integrating western interventions with traditional beliefs and practice. Soc Sci Med. 2005 Jan;60(1):179–89.
58. Metzler J, Saw T, Nono D, Kadondi A, Zhang Y, Leu CS, et al. Improving adolescent mental health and protection in humanitarian settings: longitudinal findings from a multi-arm randomized controlled trial of child-friendly spaces among South Sudanese refugees in Uganda. J Child Psychol Psychiatry. 2023 Jun;64(6):907-917. doi:10.1111/jcpp.13746.
59. Momotaz H, Ahmed H, Jalal Uddin MM, et al. Implementing the Mental Health Gap Action Programme in Cox’s Bazar, Bangladesh. Intervention. 2019 May 1;17(2):243–51.
60. Murray LK, Tol W, Jordans M, et al. Dissemination and implementation of evidence based, mental health interventions in post conflict, low resource settings. Intervention (Amstelveen). 2014 Dec;12(Suppl 1):94-112.
61. Quosh C. Comprehensive mental health and psychosocial support case management and indicative care pathways within humanitarian settings. Intervention. 2016 Nov;14(3):281–92.
62. Khan YS, Khan AW, Alabdulla M. Mental health considerations of a humanitarian crisis: Identification of needs and delivery of services to Afghan child and adolescent refugees in Qatar. Asian J Psychiatr. 2022 Jul;73:103128.
63. Sonderegger R, Rombouts S, Ocen B, et al. Trauma rehabilitation for war-affected persons in northern Uganda: a pilot evaluation of the EMPOWER programme. Br J Clin Psychol. 2011 Sep;50(3):234-49.
64. Sullivan J, Thorn N, Amin M, et al. Using simple acupressure and breathing techniques to improve mood, sleep and pain management in refugees: a peer-to-peer approach in a Rohingya refugee camp. Intervention. 2009;17(2), 252-258.
65. Tarannum S, Elshazly M, Harlass S, et al. Integrating mental health into primary health care in Rohingya refugee settings in Bangladesh: Experiences of UNHCR. Intervention. 2019 May 1;17(2):130–9.
66. Tol WA, Leku MR, Lakin DP, et al. Guided self-help to reduce psychological distress in South Sudanese female refugees in Uganda: a cluster randomised trial. Lancet Glob Health. 2020 Feb;8(2):e254-e263.
67. Amsalu R, Schulte-Hillen C, Garcia DM, et al. Lessons Learned From Helping Babies Survive in Humanitarian Settings. Pediatrics. 2020 Oct;146(Suppl 2):S208-S217.
68. Ayoya MA, Golden K, Ngnie-Teta I, et al. Protecting and improving breastfeeding practices during a major emergency: lessons learnt from the baby tents in Haiti. Bull World Health Organ. 2013 Aug 1;91(8):612-7.
69. Azad F, Rifat MA, Manir MZ, et al. Breastfeeding support through wet nursing during nutritional emergency: A cross sectional study from Rohingya refugee camps in Bangladesh. PLoS One. 2019 Oct 2;14(10):e0222980.
70. Barua M, Saha A, Chowdhury S, Chowdhury S, Sajow SH, Sarker M. Implementation of a community-based referral project to improve access to emergency obstetric and newborn care in Rohingya population during COVID-19 pandemic in Bangladesh. BMJ Innov. 2022 Sep 29;8(4):247-254. doi:10.1136/bmjinnov-2021-000831.
71. Carrara VI, Stuetz W, Lee SJ, et al. Longer exposure to a new refugee food ration is associated with reduced prevalence of small for gestational age: results from 2 cross-sectional surveys on the Thailand-Myanmar border. Am J Clin Nutr. 2017 Jun 1;105(6):1382–90.
72. Dozio E, Le Roch K, Bizouerne C. Baby friendly spaces: an intervention for pregnant and lactating women and their infants in Cameroon. Intervention. 2020 May 1;18(1):78–84.
73. Purdin S, Khan T, Saucier R. Reducing maternal mortality among Afghan refugees in Pakistan. Int J Gynaecol Obstet. 2009 Apr;105(1):82-5.
74. Morris J, Jones L, Berrino A, et al. Does combining infant stimulation with emergency feeding improve psychosocial outcomes for displaced mothers and babies? A controlled evaluation from northern Uganda. Am J Orthopsychiatry. 2012 Jul;82(3):349-57.
75. Mullany LC, Lee CI, Paw P, et al. The MOM Project: delivering maternal health services among internally displaced populations in eastern Burma. Reprod Health Matters. 2008 May;16(31):44-56.
76. Rijken MJ, Lee SJ, Boel ME, et al. Obstetric ultrasound scanning by local health workers in a refugee camp on the Thai-Burmese border. Ultrasound Obstet Gynecol. 2009 Oct;34(4):395-403.
77. Sami S, Kerber K, Tomczyk B, et al. "You have to take action": changing knowledge and attitudes towards newborn care practices during crisis in South Sudan. Reprod Health Matters. 2017 Nov;25(51):124-139.
78. Sami S, Amsalu R, Dimiti A, et al. An analytic perspective of a mixed methods study during humanitarian crises in South Sudan: translating facility- and community-based newborn guidelines into practice. Confl Health. 2021 Dec 1;15(1):1–6.
79. Sarker M, Saha A, Matin M, et al. Effective maternal, newborn and child health programming among Rohingya refugees in Cox's Bazar, Bangladesh: Implementation challenges and potential solutions. PLoS One. 2020 Mar 26;15(3):e0230732.
80. Talley LE, Boyd E. Challenges to the programmatic implementation of ready to use infant formula in the post-earthquake response, Haiti, 2010: a program review. PLoS One. 2013 Dec 31;8(12).
81. Alemu T, Bezabih B, Amsalu A, Hassen E, Haile M, Abite M. Health and nutrition emergency response among internally displaced persons at Ranch collective site, Chagni, Ethiopia: The role of emergency operation center, lessons from the field, and way forwards. Front Public Health. 2022 Sep 15;10:926551. doi:10.3389/fpubh.2022.926551.
82. Ansbro É, Homan T, Qasem J, et al. MSF experiences of providing multidisciplinary primary level NCD care for Syrian refugees and the host population in Jordan: an implementation study guided by the RE-AIM framework. BMC Health Serv Res. 2021 Apr 26;21(1):381.
83. Ansbro É, Masri S, Prieto-Merino D, Willis R, Aoun Bahous S, Molfino L, et al. Fixed dose combination drugs for cardiovascular disease in a prolonged humanitarian crisis in Lebanon: an implementation study. BMJ Open. 2023 Jan 25;13(1):e063668. doi:10.1136/bmjopen-2022-063668.
84. Bile KM, Hafeez A, Kazi GN, et al. Protecting the right to health of internally displaced mothers and children: the imperative of inter-cluster coordination for translating best practices into effective participatory action. East Mediterr Health J. 2011 Dec;17(12):981-9.
85. Bile KM, Shadoul AF, Raaijmakers H, et al. Learning through crisis: development and implementation of a health cluster strategy for internally displaced persons. East Mediterr Health J. 2010;16 Suppl:S82-90.
86. Ehiri JE, Gunn JK, Center KE, et al. Training and deployment of lay refugee/internally displaced persons to provide basic health services in camps: a systematic review. Glob Health Action. 2014 Oct 1;7:23902.
87. Kayali M, Moussally K, Lakis C, et al. Treating Syrian refugees with diabetes and hypertension in Shatila refugee camp, Lebanon: Médecins Sans Frontières model of care and treatment outcomes. Confl Health. 2019 Apr 2;13(1).
88. Mahn M, Maung C, Oo EK, et al. Multi-level partnerships to promote health services among internally displaced in eastern Burma. Glob Public Health. 2008;3(2):165-86.
89. Murphy A, Willis R, Ansbro É, et al. Implementation of fixed-dose combination therapy for secondary prevention of atherosclerotic cardiovascular disease among Syrian refugees in Lebanon: a qualitative evaluation. BMC Health Serv Res. 2022 Jun 4;22(1):744.
90. Sethi S, Jonsson R, Skaff R, et al. Community-Based Noncommunicable Disease Care for Syrian Refugees in Lebanon. Glob Health Sci Pract. 2017 Sep 28;5(3):495-506.
91. Shortall CK, Glazik R, Sornum A, et al. On the ferries: the unmet health care needs of transiting refugees in Greece. Int Health. 2017 Sep 1;9(5):272-280.
92. Sibai AM, Najem Kteily M, Barazi R, et al. Lessons learned in the provision NCD primary care to Syrian refugee and host communities in Lebanon: the need to 'act locally and think globally'. J Public Health (Oxf). 2020 Aug 18;42(3):e361-e368.
93. El-Halabi S, Khader YS, Khdeir MA, Hanson C, Alfvén T, El-Khatib Z. Children Immunization App (CIMA): A Non-randomized Controlled Trial Among Syrian Refugees in Zaatari Camp, Jordan. J Prev (2022). 2023 Apr;44(2):239-252. doi: 10.1007/s10935-023-00721-7.
94. Khader YS, Maalouf W, Khdair MA, et al. Scaling the Children Immunization App (CIMA) to Support Child Refugees and Parents in the Time of the COVID-19 Pandemic: A Social Capital Approach to Scale a Smartphone Application in Zaatari Camp, Jordan. J Epidemiol Glob Health. 2022 Mar;12(1):7-12.
95. Klabbers RE, Muwonge TR, Pham P, Mujugira A, Vinck P, Borthakur S, et al. Leveraging interactive voice response technology to mitigate COVID-19 risk in refugee settlements in Uganda: Lessons learned implementing "Dial-COVID" a toll-free mobile phone symptom surveillance and information dissemination tool. PLoS One. 2023 Jan 23;18(1):e0279373. doi:10.1371/journal.pone.0279373.
96. Lyles E, Paik K, Kiogora J, Hussein H, Cordero Morales A, Kiapi L, et al. Adoption of Electronic Medical Records for Chronic Disease Care in Kenyan Refugee Camps: Quantitative and Qualitative Prospective Evaluation. JMIR Mhealth Uhealth. 2023 Oct 5;11:e43878. doi:10.2196/43878.
97. McEwen FS, El Khatib H, Hadfield K, Pluess K, Chehade N, Bosqui T, et al. Feasibility and acceptability of phone-delivered psychological therapy for refugee children and adolescents in a humanitarian setting. Confl Health. 2024 Jan 13;18(1):7. doi:10.1186/s13031-023-00565-2.
98. Mitchell-Gillespie B, Hashim H, Griffin M, et al. Sustainable support solutions for community-based rehabilitation workers in refugee camps: piloting telehealth acceptability and implementation. Global Health. 2020 Sep 15;16(1):82.
99. O'Laughlin KN, Xu A, Greenwald KE, et al. A cohort study to assess a communication intervention to improve linkage to HIV care in Nakivale Refugee Settlement, Uganda. Glob Public Health. 2021 Dec;16(12):1848-1855.
100. Rossi L, Materia E, Hourani A, et al. Design and implementation of a hospital information system for the Palestine Red Crescent Society in Lebanon. East Mediterr Health J. 2009 May-Jun;15(3):738-46.
101. Sampson S, Oni F, Ayodeji O, Oluwatola T, Gab-Deedam S, Adenipekun O, et al. Addressing barriers to accessing family planning services using mobile technology intervention among internally displaced persons in Abuja, Nigeria. AJOG Glob Rep. 2023 Jul 2;3(3):100250. doi:10.1016/j.xagr.2023.100250.
102. Shaikh MA. Nurses' use of global information systems for provision of outreach reproductive health services to internally displaced persons. Prehosp Disaster Med. 2008 May-Jun;23(3):s35-8.
103. Wilton KS, Murphy KM, Mahmud A, Azam S, Habib A, Ibrahim I, et al. Adapting Reach Up and Learn in Crisis and Conflict Settings: An Exploratory Multiple Case Study. Pediatrics. 2023 May 1;151(Suppl 2):e2023060221K. doi:10.1542/peds.2023-060221K.
104. Beeman A, Kwesiga J, Ippoliti N, Bhandari T, Pandya G, Acam FA, et al. Using human-centered design to co-design dedicated menstrual health spaces with people who menstruate in Bidi Bidi refugee settlement, Uganda: Learnings for further adaptation and scale in humanitarian settings. BMC Womens Health. 2023 Jun 20;23(1):319. doi:10.1186/s12905-023-02421-0.
105. Curry DW, Rattan J, Nzau JJ, et al. Delivering high-quality family planning services in crisis-affected settings I: program implementation. Glob Health Sci Pract. 2015 Feb 4;3(1):14-24.
106. Curry DW, Rattan J, Huang S, et al. Delivering high-quality family planning services in crisis-affected settings II: results. Glob Health Sci Pract. 2015 Feb 4;3(1):25-33.
107. Fetters T, Rubayet S, Sultana S, et al. Navigating the crisis landscape: engaging the ministry of health and United Nations agencies to make abortion care available to Rohingya refugees. Confl Health. 2020 Jul 23;14:50.
108. O'Connell KA, Hailegebriel TS, Garfinkel D, Durham J, Yakob B, Kassaw J, et al. Meeting the Sexual and Reproductive Health Needs of Internally Displaced Persons in Ethiopia's Somali Region: A Qualitative Process Evaluation. Glob Health Sci Pract. 2022 Oct 31;10(5):e2100818. doi:10.9745/GHSP-D-21-00818.
109. Von Roenne F, Kollie S, Swaray Y, et al. Reproductive health services for refugees by refugees: an example from Guinea. Disasters. 2010 Jan;34(1):16–29.
110. Warren E, Post N, Hossain M, et al. Systematic review of the evidence on the effectiveness of sexual and reproductive health interventions in humanitarian crises. BMJ Open. 2015 Dec 18;5(12):e008226.
111. Ahmed M, Whitestone N, Patnaik JL, et al. Burden of eye disease and demand for care in the Bangladesh Rohingya displaced population and host community: A cohort study. PLoS Med. 2020 Mar 31;17(3):e1003096.
112. Vincent JE. Simple spectacles for adult refugees on the Thailand-Burma border. Optom Vis Sci. 2006 Nov;83(11):803-10.
113. Moreau P, Ismael S, Masadeh H, et al. 3D technology and telemedicine in humanitarian settings. Lancet Digit Health. 2020 Mar;2(3):e108-e110.
114. Sechriest VF 2nd, Lhowe DW. Orthopaedic care aboard the USNS Mercy during Operation Unified Assistance after the 2004 Asian tsunami. A case series. J Bone Joint Surg Am. 2008 Apr;90(4):849-61.
115. Del Cacho ME, Lamin-Abdi M, Masoud-Mohamed M, et al. Pharmaceutical humanitarian assistance in the establishment of a drug compounding laboratory in a refugee camp. Farmacia Hospitalaria. 2021 Sep 1;45(5):262–7.
116. Rah JH, dePee S, Kraemer K, et al. Program experience with micronutrient powders and current evidence. J Nutr. 2012 Jan;142(1):191S-6S.
