# Supplementary material for: Innovative strategies and implementation science approaches for health delivery among migrants in humanitarian settings: A scoping review
Source: PLOS Glob Public Health. 2024 Dec 2;4(12):e0003514. doi: 10.1371/journal.pgph.0003514 (PMC11611092; doi:10.1371/journal.pgph.0003514)
Supplement: Attachment — Submitted filename: PLOS Global Public Health June 2024 Review1.pdf [file pgph.0003514.s004.pdf]

Independent Review Report

Study Title
Innovative strategies and implementation science approaches for health delivery among migrants in humanitarian settings: a scoping review

Focus
The focus of the study was:
“This scoping review evaluates implementation science in health services for forced migrants in humanitarian settings”.

Summary of main findings/outcomes of the study

- “More than 90% of studies collectively identified 35 implementation strategies, the most common being capacity building (44%), stakeholder engagement (35%), and information dissemination (38%), and feedback mechanisms (25%). Only 10 studies used formal implementation models, with RE-AIM (n=3) and Intervention mapping (n=2) being most frequent”. In addition, the scoping review reports “that common implementation science strategies were often utilized to deliver health care to migrants in humanitarian settings. Though studies came from diverse regions, populations, and topic areas, determinants (barriers and facilitators) and implementation strategies were well described, shared, and could be defined and categorized among the studies”.

Methodology
The methodology is well stated and presented in the manuscript.

Manuscript methods, data and analysis align with the study conclusion.
As reported in this manuscript, the methods used, the data collection, mode of data analysis and discussion of findings are adequately explained to support the conclusions reached.

Replicability
The methods, data collection and data analysis in this manuscript are explained and can be reproduced following the PRISMA-ScR.

Statistical review
Not applicable.

Scholarly contributions
This study contributes to the global literature on “Prevalence of Hypertension and associated factors”.

The strengths and limitations of the study
Strengths

- The search through eight databases provides a wide range for extracted materials.

Limitations

- The study limitations are well presented.

Manuscript is worth publishing.
This manuscript is worth to be published after all minor corrections are done by the authors.

Valid arguments supported by evidence.
This manuscript's arguments are well supported by the evidence reported in the scoping review.

Ethical concerns
Not applicable.

Minor Issues

- Exclusion criteria: Non-English Language published materials were excluded from the scoping review.
- Table 1 should be put in Landscape form. This table is essential. It can be expanded to include **type of study**.
- **GENERAL OBSERVATION**
- The manuscript should be double-checked again for GRAMMAR mistakes.

Independent Reviewer Decision: Accept the paper for publication after MINOR revisions and queries have been addressed.
