# Supplementary material for: Innovative strategies and implementation science approaches for health delivery among migrants in humanitarian settings: A scoping review
Source: PLOS Glob Public Health. 2024 Dec 2;4(12):e0003514. doi: 10.1371/journal.pgph.0003514 (PMC11611092; doi:10.1371/journal.pgph.0003514)
Supplement: Attachment — Submitted filename: Point by point response.docx [file pgph.0003514.s003.docx]

Dear Editorial Office:

We appreciate your recommended revisions to our manuscript, “Innovative strategies and implementation science approaches for health delivery among migrants in humanitarian settings: a scoping review.” We agree with the recommended changes and have incorporated them into our manuscript. As instructed, we submitted the revised manuscript as a new submission to PLOS Global Public Health and have included a point-by-point response below in italicized font. We look forward to receiving your feedback and the chance to be considered for publication.

**Editor comments:**

Dear Dr. Reynolds,

Thank you for submitting your manuscript for review by PLOS Global Public Health. After careful consideration, we have decided that your manuscript does not meet our standards for systematic and scoping reviews. We are therefore rejecting your manuscript. PLOS Global Public Health considers systematic reviews and meta-analyses for publication; however, these submissions must address a clear and defined research question; conduct a systematic and comprehensive literature review; and use clearly reported, reproducible, and systematic methods to identify, select, and critically appraise relevant research. Our author guidelines for systematic reviews are at <https://journals.plos.org/globalpublichealth/s/submission-guidelines#loc-systematic-reviews-and-meta-analyses>. In the case of your submission, we are concerned that the literature review was completed in October 2022. Systematic and scoping reviews should provide an up-to-date overview of the research topic covered. Since the literature search was completed over a year ago, we are concerned that this study does not meet this requirement. As a result of these concerns, we cannot consider the manuscript for publication at PLOS Global Public Health at this time. However, if you are able to address the concerns discussed above, we may be able to consider a revised version of the manuscript. This would however require a substantial amount of work and thus, would be considered as a new submission. If you are able to revise the manuscript to respond to the concerns noted above, please refer to the original submission in the covering letter and accompany the submission by a rebuttal letter that responds to the comments made on the original manuscript.

**Author response:**

*Following these recommendations, we reconducted our original search strategy to include articles from October 2022-February 2024. This resulted in the identification of 1,222 new abstracts, of which 24 were included following full-text review. These studies were aggregated to our existing included studies, for a new total of 116 studies included in the scoping review. We updated our methods and results sections to include these revisions. These revisions included for Tables 1, 2, and 3, as well as Appendix 2 and the reference to our search database which will allow readers to access the original data including search strategy and initial findings. We have included the specific in-text changes to the methods section below:*

*Methods, line 154: “A search of the literature was conducted by a health sciences informationist (GKR) in October 2022 and a search update was implemented in February 2024.”*

*Methods, line 165: “Search results were limited to English or Spanish articles published from 2000 to February 2024 in six of the eight databases. Language limits were not used in the search of the CENTRAL; and language or year limits were not required for the search implemented in the Cochrane Covid-19 Study Register. A total number of 10,857 citations were exported to the citation manager EndNote (Clarivate Analytics) for processing and removal of duplicate articles. After removal of duplicates using a variation on the Bramer method [24], 7,795 citations were exported to the evidence synthesis screening tool Rayyan [25] for assessment and initial screening.”*

*Methods, line 178: “The literature search strategies and citation files are available at https://dx.doi.org/10.7302/22500 [26].”*
