# Supplementary material for: Innovative strategies and implementation science approaches for health delivery among migrants in humanitarian settings: A scoping review
Source: PLOS Glob Public Health. 2024 Dec 2;4(12):e0003514. doi: 10.1371/journal.pgph.0003514 (PMC11611092; doi:10.1371/journal.pgph.0003514)
Supplement: Attachment — Submitted filename: Response to reviewers.docx [file pgph.0003514.s005.docx]

Dear Dr. Onuigbo Atulomah:

We’re very pleased with the response to our manuscript and the thoughtful recommendations from the reviewers. Below we have included a point-by-point response to each review, with our response to each reviewer in italicized font and references to the in-text corrections. We are convinced that this is now a better manuscript and look forward to your review of these revisions.

Thank you for your continued consideration of our manuscript for publication in *PLOS Global Public Health.*

Sincerely,

Christopher Reynolds, MD, MS

Department of Surgery

University of Michigan Medicine

Response to reviewers:

Reviewer #1: The article is timely, well researched and excellently written. The objectives, methods and findings are well stated and justified. It is applicable to ongoing humanitarian efforts in different parts of the world, especially in resource-prive countries which strikingly are the worst hit by emergencies that lead to forced migration of their populations. I agree with the conclusion reached by the authors that "Implementation science provides an exciting opportunity for more effectively planning, implementing, and evaluating health services programming for migrants in humanitarian contexts".

*Author response: Thank you for your kind words and taking the time to review our manuscript.*

Reviewer #2: Thank you for inviting me to review this manuscript. I believe that it will make significant contributions in the field of the subject area. The article is well written. The title, abstract, methodology, results and conclusion all meet the requirements.

*Author response: Thank you for your kind words and taking the time to review our manuscript.*

Reviewer #3: • Exclusion criteria: Non-English Language published materials were excluded from the scoping review.

*Author response: Thank you for taking the time to review our manuscript. We have added this recommendation into our exclusion criteria, in that we excluded non-English or Spanish language published materials:*

*Methods, page 8, line 197: “Studies which were not published in English or Spanish were excluded from this scoping review.”*

• Table 1 should be put in Landscape form. This table is essential. It can be expanded to include type of study.

*Author response: Thank you for this helpful recommendation. We have updated Table 1 with study type for each reference. We also change the layout to Landscape form as recommended.*

*Results, page 12-25, see Table 1.*
